# Antibiotic Resistance in Recreational Waters: State of the Science

**DOI:** 10.3390/ijerph17218034

**Published:** 2020-10-31

**Authors:** Sharon P. Nappier, Krista Liguori, Audrey M. Ichida, Jill R. Stewart, Kaedra R. Jones

**Affiliations:** 1 U.S. Environmental Protection Agency, Office of Water, Office of Science and Technology, 1200 Pennsylvania Avenue, NW, Washington, DC 20460, USA; Krista.Liguori@gmail.com; 2 ICF, LLC, 9300 Lee Highway, Fairfax, VA 22031, USA; Audrey.Ichida@icf.com (A.M.I.); kaedra.jones@icf.com (K.R.J.); 3 1301 Michael Hooker Research Center, Gillings School of Global Public Health, University of North Carolina, Chapel Hill, NC 27599, USA; Jill.Stewart@unc.edu

**Keywords:** antibiotic resistance, antimicrobial resistant bacteria, antimicrobial resistant genes, recreational exposures, aquatic microbial community, aquatic microbiome, wastewater, human health risk, ambient water, surface water

## Abstract

Ambient recreational waters can act as both recipients and natural reservoirs for antimicrobial resistant (AMR) bacteria and antimicrobial resistant genes (ARGs), where they may persist and replicate. Contact with AMR bacteria and ARGs potentially puts recreators at risk, which can thus decrease their ability to fight infections. A variety of point and nonpoint sources, including contaminated wastewater effluents, runoff from animal feeding operations, and sewer overflow events, can contribute to environmental loading of AMR bacteria and ARGs. The overall goal of this article is to provide the state of the science related to recreational exposure and AMR, which has been an area of increasing interest. Specific objectives of the review include (1) a description of potential sources of antibiotics, AMR bacteria, and ARGs in recreational waters, as documented in the available literature; (2) a discussion of what is known about human recreational exposures to AMR bacteria and ARGs, using findings from health studies and exposure assessments; and (3) identification of knowledge gaps and future research needs. To better understand the dynamics related to AMR and associated recreational water risks, future research should focus on source contribution, fate and transport—across treatment and in the environment; human health risk assessment; and standardized methods.

## 1. Introduction

The increasing rise of antimicrobial resistance (AMR) is one of the greatest threats to human health in the 21st century [1,2,3]. Today, antibiotics remain important for treating both minor and life-threatening bacterial infections. They are also essential for the prevention of infection following surgical procedures and in the administration of treatments that depress the immune system, including the administration of chemotherapeutic agents to cancer patients [4,5]. Patients with infections caused by resistant bacteria are at increased risk of adverse clinical outcomes and consume more health-care resources than patients infected with non-resistant or sensitive strains of the same bacteria [6]. For example, it is estimated that death is 64% more likely in people infected with methicillin-resistant *Staphylococcus aureus* (MRSA) compared to people infected with a sensitive form of the bacteria [6]. As pathogens develop resistance to multiple antibiotics, there is a risk that treatment failures using last resort antibiotics will occur more frequently. Antibiotics and antimicrobials also play crucial roles in crop production, animal husbandry, and aquaculture. Overuse, and subsequent increased resistance, within our food supply has the potential to greatly impact our global food supply and contribute to the spread of AMR globally [7,8]. Because of the importance of antibiotics for the protection of public health and food production, there is increasing interest in understanding the environmental sources, reservoirs, and exposure pathways of antibiotic resistance. This represents an emerging field known as environmental antimicrobial resistance (“environmental AMR”).

An increasing body of research identifies the environment as not only a recipient of drug-resistant bacteria, but as a reservoir and source of resistance genes [9,10,11,12,13]. AMR bacteria and associated genes (also commonly referred to as “antibiotic resistant” or “drug resistant”) have therefore become an emerging concern regarding the protection of human health during recreational activities in ambient surface waters [14,15,16,17], yet it remains unclear if exposure to microorganisms with ARGs presents a risk to recreators. 

Resistance to traditional fecal indicator bacteria (FIB), such as *Escherichia coli* (*E. coli*) and enterococci, and to bacterial pathogens are of interest in ambient recreational waters. Bacterial pathogens, include but are not limited to, those in the Enterobacteriaceae family (e.g., pathogenic *E. coli*, *Klebsiella* spp., *Salmonella* spp., *Shigella* spp., and *Yersinia pestis*) and *Staphylococcus aureus* (specifically MRSA), which have all been detected in ambient waters [15,18,19,20]. Overall, the presence of AMR bacteria and ARGs in surface water is well established [15,16,17,18,21,22,23,24,25,26,27,28,29,30,31]. However, direct comparisons between these studies are difficult as the geography, AMR bacteria and ARGs selected for evaluation, sources of fecal impacts, and methods of determining resistance are highly variable between studies [27,32]. The presence of AMR bacteria and ARGs in recreational waters suggests the potential for exposure to resistant microorganisms during swimming and similar recreational water activities [25,28,31,33]. 

Both point and nonpoint sources of fecal contamination, and even naturally occurring bacteria, are potential origins of AMR bacteria and ARGs in recreational waters [9,34,35,36]. Many countries have developed water quality recommendations for FIB to protect surface waters that are designated for primary contact recreation activities from pathogens associated with human fecal contamination [37,38,39]. In the United States (U.S.), these water quality recommendations are also used to derive point source discharge permits, called National Pollution Discharge Elimination System (NPDES) permits, used to limit discharge of human pathogens into waterbodies from point sources. However, surface water recommendations and associated permits do not specifically address the presence of AMR bacteria and ARGs. Although processes commonly used at wastewater treatment plants (WWTPs) are designed to reduce, remove, or inactivate pathogenic bacteria and associated FIB, AMR bacteria and ARGs have been detected in effluent shortly before its discharge to nearby surface waters [33,40,41]. Bacteriophages carrying ARGs have also been detected in WWTP effluent, indicating the genes’ ability to survive removal processes and possible presence in discharged effluent [42,43]. 

The goal of this review article is to provide the state of the science related to recreational water exposure and AMR bacteria and ARGs. Objectives of this review are to (1) describe potential sources of antibiotics, AMR bacteria, and ARGs in recreational waters as documented in the available literature; (2) discuss what is known about human recreational exposures to AMR bacteria and ARGs using findings from epidemiological studies and exposure assessments; and (3) identify knowledge gaps and future research needs.

## 2. Mechanisms of Gene Persistence and Transfer in Surface Waters

Antibiotic resistance and resistance genes are intrinsic microbial attributes that existed long before the first antibiotic was utilized by humans in the early 1900s [13,44,45,46]. AMR bacteria and ARGs can be found in pristine environments, considered unimpacted by anthropogenic activities [47,48,49,50,51,52]. A broad range of antibiotics are naturally produced by soil and waterborne microorganisms including bacteria, fungi, and actinomycetes [13,53]. Historically, microorganisms likely used natural antibiotics as a method of self-defense against competing microorganisms or for signaling with nearby microorganisms influenced by environmental pressures and maintained through genetic mechanisms [9,54].

Bacteria have proven adept at developing resistance to antibiotics produced by humans [53,55]. Humans have produced and either directly or indirectly released millions of metric tons of antibiotics into the environment in the last 50 years via applications ranging from human and veterinary medicine, personal care products, and commercial food animal production [33,53,56,57]. Use of antibiotics produces a selective pressure on bacteria, favoring resistant bacteria that then multiply or share resistance genes through horizontal gene transfer (HGT) [55,57]. HGT occurs in one of three ways: (1) uptake of genetic material from the environment (i.e., transformation); (2) direct transfer of genetic material from one cell to another (i.e., conjugation); or (3) movement of genetic material from one cell to another via a bacteriophage vector (i.e., transduction) or other cell-free DNA mechanisms [55,58]. A diversity of mobile genetic element types facilitates HGT, including plasmids, integrons, and prophages [32,33], all of which allow for sharing of resistance among bacteria. Hotspots for HGT and AMR selection are created by a combination of conditions favorable for bacterial growth and genetic transfer, including high cell densities, availability of nutrients, and high concentrations of pollutants [59,60]. Additionally, multispecies microbial communities, referred to as biofilms, are thought to be important ARG reservoirs that proliferate AMR bacteria and ARGs in aquatic environments [61].

While resistance genes provide adaptive advantages to bacteria when antibiotic exposure does occur, ARGs can also be carried and shared without the selective pressure of antibiotic exposure. For example, ARGs can be transferred within a bacterial population when maintenance of the gene does not affect a cell’s ability to thrive in its environment [62]. Additionally, AMR and heavy metal resistance genes often co-occur on the same mobile genetic elements (e.g., plasmids). Thus, exposure to heavy metals can exert a selective pressure, resulting in a co-selection and propagation of mobile genetic elements carrying ARGs, even in the absence of antibiotics [33,63,64,65,66,67,68,69,70,71]. In addition, resistance can be acquired through spontaneous mutations [30].

Resistant genes can move between pathogenic and nonpathogenic microorganisms [33,72], and either could be considered useful for monitoring AMR in ambient waters. For example, *Aeromonas* spp., which can be pathogenic and indigenous, may play a role in disseminating ARGs because they (1) naturally produce three β-lactamases; (2) contain mobile genetic elements that can be transferred to other human pathogens [73]; and (3) have been detected in surface waters [74,75,76,77]. Berendonk et al. [75] propose the use of *Aeromonas* spp. as a surface water indicator of ARGs when fecal contamination is not expected in environmental samples. Berendonk et al. [75] also propose *E. coli* and enterococci as surface water indicators of ARGs because they are currently used to monitor microbiological water quality in recreational waters and are well characterized in terms of acquired antibiotic resistance [40,78,79,80]. Ingestion of water containing antibiotic-resistant *E. coli* during recreational activities can be associated with gut colonization by these bacteria [16,17,81]. For example, extended-spectrum β-lactamase (ESBL)-producing *E. coli* have been isolated from recreational water in the United Kingdom, and gut colonization with ESBL-*E. coli* was confirmed among surfers recreating in those waters. 

## 3. Anthropogenic Sources of Antibiotic Resistance in Recreational Waters

**Figure 1:**
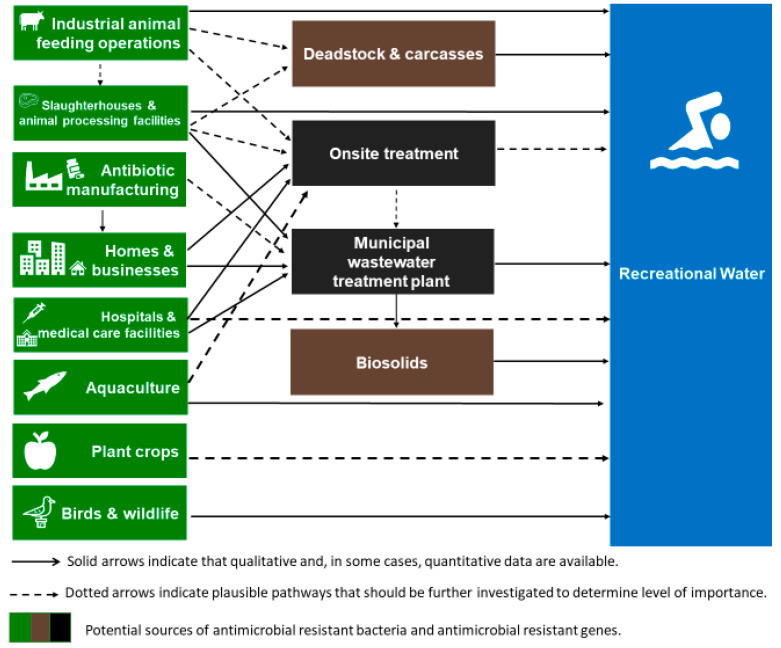
Potential sources of antimicrobial resistant (AMR) bacteria and antimicrobial resistant genes (ARGs) contributing to recreational surface water exposures.

Point and nonpoint sources contribute to the presence and proliferation of AMR bacteria and ARGs in surface waters [9,34,35,36]. Figure 1 shows the known sources of resistance contaminants and how they could potentially contribute to recreational water exposures to AMR bacteria and ARGs [34,36,73,82]. Feasible pathways of movement for AMR bacteria and ARGs vary for the different potential sources, depending on factors including, but not limited to, local regulations and industrial practices. For example, slaughterhouses and animal processing facilities are subject to different treatment regulations, depending on the country. Thus, the pathway(s) for this potential source varies by geographical location. Availability of data for different countries and local situations also varies. Within Figure 1, onsite treatment technologies and approaches vary and might include lagoons and runoff ponds at industrial animal feeding operations; septic systems for small slaughterhouse, homes, or businesses; or more advanced biological treatment with disinfection at larger facilities, and tertiary treatment with ultraviolet radiation or chlorine disinfection at hospitals and medical care facilities.

### 3.1. Untreated Wastewater

Much of the research in the emerging field of environmental AMR has focused on wastewater as a source of AMR bacteria and ARGs to water. Untreated wastewater influent (or raw sewage) contains concentrated levels of pathogenic and nonpathogenic microbes [83,84] and antibiotics [85]. Wastewater and raw sewage can enter surface waters via combined sewer and sanitary sewer overflows [86,87]. EPA estimates that there are between 23,000 and 75,000 sanitary sewer overflows per year (not including sewage backups into buildings) in the U.S. [88]. During wet weather, WWTPs with combined sewers receiving stormwater may experience flow rates that are higher than their design capacity, and thus blended water can be released without full treatment [89]. Honda et al. [90] estimated the annual concentrations of AMR *E. coli* discharged from combined sewer overflows using the abundance of AMR, *E. coli* concentrations, and the flow rate of combined sewage and secondary treatment effluents. Annual concentrations of AMR *E. coli* discharged from combined sewer overflows were approximately 5000 times higher than from WWTP effluent [90]. Additionally, aging infrastructure can result in the leakage of raw sewage from pipes into the ground, potentially contaminating groundwater supplies, wells, and source waters [91,92,93]. Bacteria contained in sewage leachate may further lead to the spread of environmental AMR [94]. 

There have been many studies evaluating AMR in raw sewage (or influent) of residential areas, hospitals, and municipal WWTPs [40,95,96,97]. A global monitoring study of urban sewage evaluated systematic differences in the abundance and diversity of ARGs [98]. Significant regional differences were observed, and these differences correlated with socioeconomic factors more than antibiotic use [98]. Two clusters of ARG abundance were identified: one cluster consisted of high-income countries in Europe, North America, and Oceania and the second cluster consisted of low- and middle-income countries in Africa, Asia, and South America [98]. In high-income countries in Europe, North America, and Oceania, a high abundance of a limited number of ARGs encoding macrolide resistance genes was detected [98]. In some low- and middle-income countries in Africa, Asia, and South America, a high abundance of diverse ARGs from different drug classes was detected [98]. The most divergent distribution of ARGs was found in India, Vietnam, and Brazil, suggesting these countries could be hotspots for the emergence of new mechanisms of AMR [98]. 

The occurrence of resistance genes in raw wastewater can vary both geographically and temporally. Pärnänen et al. [99] studied the urban sewage influent resistome (229 resistance genes and 25 mobile genetic elements in 168 wastewater samples) for seven countries (Portugal, Spain, Ireland, Cyprus, Germany, Finland, and Norway). In this study, most of the ARGs detected in the influents corresponded to genes resistant to first-generation antibiotics, with widespread environmental distribution. All influent samples contained genes conferring resistance to aminoglycosides, β-lactams, macrolide–lincosamide–streptogramin B, sulfonamides, tetracyclines, and multidrug resistance. The genetic signatures for elements involved in gene transfer and recombination were also present in all influent samples. The relative abundance of resistant gene families and gene transfer determinants was higher in the countries with higher antibiotic consumption. Joseph et al. [60] collected sewage samples over 6 months across five New York City boroughs and detected seven ARGs in all samples. Levels of five of the seven ARGs fluctuated over the time period, indicating a potential seasonal pattern that could be the basis for future research [60]. The source of ARGs could not be determined as multiple sources contributed to the sewage samples, but ARG occurrence likely varies temporally with infection rates, prescription use, and other factors.

#### Medical Waste

Originating in hospitals and other healthcare facilities, pathogenic bacteria with resistance against all or almost all existing antibiotic treatments are of increasing concern [100]. Antibiotics, pharmaceutical residues, and AMR bacteria and genes have been detected in hospital wastewaters: (1) treated by municipal WWTPs (i.e., hospital is an indirect discharger) [101,102]; (2) treated by onsite treatment systems [97,103]; and (3) with no treatment at all prior to discharge (i.e., direct discharger) to surface water [24,97,102]. Antibiotics and antibiotic residues can enter municipal wastewater influent through pharmaceutical disposal, urine and feces from patients treated with pharmaceuticals and from antimicrobials used for cleaning surfaces or washing hands or clothes [104,105,106,107]. Raw sewage may be blended with pre-treated and/or raw medical waste, creating a hotspot for the transfer of ARGs [40]. 

Divyashree et al. [97] analyzed samples of untreated wastewater collected from a hospital in India. A high level of resistance towards nalidixic acid (70 of 106 samples), cefotaxime (77 of 106 samples) and ampicillin (70 of 106 samples) was found among Gram-negative bacteria isolated from untreated effluent samples [97]. Lamba et al. [103] compared the levels of FIB, AMR bacteria, and ARGs in hospital and municipal wastewaters. The authors found an association between the levels of FIB in wastewater leaving hospitals and the concentration of carbapenem-resistant *Enterobacteriaceae* (CRE) and ESBL-resistance genes. For this study, the level of onsite treatment prior to discharge was not reported. The study also found a positive association between levels of ESBL-resistant bacteria in hospital wastewater and the relative abundance of ESBL-resistance genes, validating the assumption that more genes can lead to more phenotypically resistant bacteria [103]. The same associations and high levels of FIB, AMR bacteria, and ARGs were not observed in municipal wastewater absent of hospital waste [103]. Similarly, in a study of Swedish sewage samples, Hutinel et al. [107] reported a higher variability in resistance rates in hospital sewage than in municipal sewage, indicating that antibiotics in hospital sewage might reach concentrations capable of selecting for resistant bacteria in sewer pipes. 

Healthcare wastewaters also contain a higher-than-average concentration of pathogenic bacteria, which may share or acquire genes (via HGT) or be selected (i.e., will multiply faster than other bacteria present in wastewater due to the presence of a mutation) during wastewater pre-treatment processes [23,108]. These selective pressures increase the likelihood of detecting AMR in hospital wastewater and downstream receiving waters. For example, concentrations of ciprofloxacin were measured above the predicted no-effect concentration, the level at which there is predicted to be no adverse or beneficial effect in humans, in hospital wastewater in Switzerland prior to treatment [108]. Although some treatment methods were effective at reducing levels of antibiotics, ciprofloxacin and fluoroquinolones were still detected in final effluent [108]. Voigt et al. [109] found that clinical wastewater forms a distinct cluster concerning resistance and ciprofloxacin is a good indicator of the presence of multidrug resistant *P. aeruginosa* and ESBL-producing *Klebsiella* spp., *Enterobacter* spp., and *Citrobacter* spp. Their results highlight the role of clinical wastewater in the dissemination and development of multidrug resistance [109]. 

Pharmaceutical production facilities are another source of antibiotics in both raw sewage and surface waters and can contribute to AMR. In a national study of twenty U.S. WWTPs, influents impacted by pharmaceutical production facilities had significantly higher concentrations of 33 pharmaceuticals (of 120 tested) compared to control WWTPs not impacted by pharmaceutical production facilities [110]. The concentration of fluconazole was three orders of magnitude higher at sites impacted by pharmaceutical production facilities compared to non-impacted sites [110]. Concentrations of celecoxib, dehydronifedipine, diazepam, phenytoin, temazepam, and verapamil were two orders of magnitude higher at sites impacted by pharmaceutical production facilities (concentrations ranging from 2500 nanograms per L to 43,800 nanograms per L) compared to non-impacted sites (concentrations ranging from 5.3 nanograms per L to 320 nanograms per L) [110]. In China and India, facilities have been known to directly discharge large volumes of antibiotics to surface waters, which may result in concentrations comparable to those used to treat human infections [111,112]. For example, concentrations of 19.5 milligrams (mg) per liter (L) of oxytetracycline and 31 mg per L of ciprofloxacin have been detected in the finished effluent of pharmaceutical manufacturing facilities in China and India, respectively [111,112].

### 3.2. Treated Wastewater

Treated WWTP effluent may act as a point source of AMR once discharged to surface waters [24,113,114,115]. Several studies have tracked the source of AMR bacteria and ARGs in surface water samples to nearby WWTPs [24,113,114,115]. Studies generally agree that while conventional wastewater treatment reduces the concentration of bacteria in water, it does not appreciably reduce the proportion of resistant bacteria [116,117,118,119]. Studies also indicate that wastewater effluent is likely to contribute resistant bacteria, including resistant and multidrug resistant *E. coli*, to aquatic environments [85,119,120,121].

Studies evaluating the prevalence of AMR in WWTP effluent indicate that wastewater treatment processes, including some secondary and tertiary treatment and disinfection processes, may not fully eliminate AMR bacteria and ARGs and that surviving bacteria retain their resistance properties [41,72,97,113,115,122,123,124,125,126,127,128,129]. Hiller et al. [72] reviewed the available literature on the levels of AMR bacteria and ARGs present following treatment with common WWTP processes, including disinfection with UV, ozone, and chlorine, to assess their efficacy. Hiller et al. [72] concluded “these findings confirm that discharge from WWTPs can result in significant contributions of AMR to the aquatic environment.” Treatment processes commonly used at WWTPs are not typically calibrated to target removal of AMR bacteria or ARGs, with concerns about persistence and transformation of both cell-associated ARGs and extracellular DNA coding for antibiotic resistance [127,128]. The effectiveness of some disinfectants, including chlorine and ozone, are impacted by dose, contact time, temperature, and water quality variables (e.g., pH, turbidity, presence of ammonia and oxidant demand) [130,131].

In the U.S., municipal waste is generally treated by primary and secondary wastewater treatment processes, with many plants employing additional disinfection steps to remove pathogens before discharging the effluent into surface waters. Depending on the designated uses of the receiving water bodies, some U.S. WWTPs disinfect effluent seasonally, rather than year-round [132]. Primary and secondary wastewater treatment processes may only partially remove pharmaceuticals, including antibiotics [108,133]. A variety of pharmaceutical molecules, including both human and veterinary antibiotics, has been detected in treated wastewater effluent [108,134], indicating that antibiotic residues may survive treatment. In a study in Wisconsin, secondary treated effluent from seven WWTPs were tested for 21 antibiotics. Six of the tested antibiotics (sulfamethazine, sulfamethoxazole, tetracycline, ciprofloxacin, erythromycin, and trimethoprim) were identified in treated effluent samples (1 to 5 per site) from these facilities [135]. The authors observed that the size of the WWTP did not impact the presence of antibiotics in effluent samples with detectable concentrations of antibiotics present in samples from WWTPs of varying sizes [135].

Some studies have shown a relative increase in AMR bacteria and ARGs in treated effluent as compared to influent [40,41,127]. Subsequently, it has been suggested that WWTPs may be hotspots for the selection, transfer, and evolution of ARGs [136,137]. Mao et al. [41] found the prevalence of 12 different ARGs was higher in secondary treated effluent than in influent. One explanation for this observation is that antimicrobials and residues present in influent may promote selection of AMR bacteria and ARGs, which remain present in effluent following application of processes to remove bacteria [40]. Selective pressure and ARG proliferation in WWTPs might be reduced by decreasing or preventing the release of antibiotics and heavy metals to sewage systems and improving technologies that support their removal in pre-treatment units [41]. There is research demonstrating that certain treatment protocols including membrane biological reactors [129] and coagulation [138] show promise for removing AMR bacteria and ARGs. There have been conflicting reports in the literature on the effect of disinfection in the removal or spread of resistance [139], likely due to differences in the disinfectants studied and the methods used to test antibiotic resistance. Interestingly, recent studies have demonstrated an increase in HGT during chlorine disinfection commonly used in conventional waste treatment [140]. The phenomenon was also observed for sunlight disinfection, but not for UV disinfection that left resistance genes unable to function regardless of repair mechanisms [141].

A WWTP that utilized advanced treatment processes (i.e., tertiary treatment using a mixed media filter consisting of anthracite coal, silica sand, and garnet followed by disinfection using sodium hypochlorite) was identified as a point source of ARGs into surface waters in Duluth-Superior Harbor [113]. A statistically significant increase in three ARGs that confer resistance to tetracycline was found in the discharged tertiary-treated effluent compared to background levels in surrounding surface waters [113]. Similar findings were reported by a Swiss study that found a 200-fold higher level of ARGs in secondary treated effluent (i.e., chemical phosphate removal process and aerobic biological treatment followed by clarification) discharged into a lake compared to reference sites in the center of the same lake [114]. In a large surveillance study of effluents from 16 WWTPs and their receiving waters across ten European countries, six of the nine ARGs were detected in all samples of effluent and their receiving waters (river water) [115]. Concentrations of ARGs in effluents were inversely correlated to the number of biological treatment steps applied by WWTPs. Additionally, Subirats et al. [142] found that in mesocosm experiments with continuous cultures, even high-quality wastewater with secondary (activated sludge) and tertiary (aluminum polychloride enriched by calcium hydroxide and anionic polyacrylamide) treatments and chlorine disinfection had undesirable effects on receiving bacterial communities in terms of composition and dissemination of ARGs. Statistically significant increases in the resistance gene pool and likelihood of gene transfer among the bacteria population in the aquatic environments were observed [142].

#### Medical Waste

Drug-resistant microbes can spread into surface water and from there into recreational waters through insufficient treatment of hospital wastes [100]. Reinthaler and colleagues [101] studied effluent from three WWTPs employing secondary treatment using activated sludge: the first receiving municipal waste, the second receiving municipal waste and water from a landfill, and the third receiving municipal waste and sewage from a hospital and a nursing home. Effluent from the WWTP receiving combined healthcare sewage with municipal waste contained higher rates of AMR in *E. coli* than effluent from WWTPs absent of wastewater from healthcare facilities [101], a finding consistent with Lamba et al. [103]. Effluents from the two facilities not receiving healthcare sewage had statistically significantly lower tetracycline resistance and statistically significantly lower ampicillin resistance than effluent from the WWTP receiving the healthcare sewage combined with municipal waste. Elevated levels of ARGs were also found in a recreational lake receiving outfalls of a WWTP (employed treatment process not reported) that receives sewage from hospitals and municipal waste [24]. High levels of multi-resistant strains of bacteria and ARGs were detected in the lake water and sediment [24]. Comparisons showed statistically significantly higher loading of AMR bacteria and ARGs from the hospital waste compared to municipal wastewater before they entered the WWTP [24]. The conventional WWTP was successful in reducing bacterial loads (78% reduction), but multi-resistant strains of AMR bacteria were preferentially selected throughout treatment, and accumulation of ARGs was observed [24].

In the U.S., most healthcare facilities discharge to municipal waste systems and are categorized as indirect dischargers, meaning waste is treated at a WWTP prior to entering surface waters [143]. Of the indirect dischargers, approximately 1500 health care facilities require pre-treatment programs to adhere to NPDES permits, depending on the state water quality standard [143]. Pre-treatment processes prevent overloading publicly owned water infrastructure with heavy loads of contaminants and may include, but are not limited to, filtration, de-gritting, and ultraviolet disinfection [100]. Indirect dischargers are also subject to regulations by the local sewer authority and local prohibitions specific to medical waste discharges. A small number of U.S. healthcare facilities (approximately 100 facilities with more than 1000 occupied beds) are considered direct dischargers. Direct dischargers are also required to adhere to NPDES permits that include limits for 5-day BOD, total suspended solids, pH, fecal coliforms, oil, and grease, as well as other EPA-approved state or tribal water quality criteria or standards that were designed to protect designated uses of surface waters, such as supporting aquatic life or recreation [143,144]. Direct dischargers, depending on their onsite treatment, have the potential to introduce AMR bacteria and ARGs into surface water. Wastewater treatment processes do not fully eliminate AMR in effluent, and surviving AMR bacteria retain their resistance properties [72].

### 3.3. Biosolids

Biosolids, the sludge generated by the treatment of sewage at WWTPs, are used as a soil amendment for agriculture, landscape, and for use in home gardens [145]. They may also be a source of antibiotics and AMR [125,129,146]. In one study, the densities of various ARGs were found to be three orders of magnitude higher in both agricultural land-applied and landfilled biosolids prior to application (class not specified) than in effluent from the same WWTPs [147]. Results indicated that use of advanced biosolids process methods (i.e., lime stabilization and anaerobic digestion) provided improved reduction of AMR bacteria and ARGs in biosolid samples when compared to conventional biosolids process methods (i.e., dewatering and gravity thickening). Other studies of thermophilic anaerobic sludge digestion also indicate that this method appears to be effective in reducing AMR bacteria and ARGs in biosolids [148,149].

Regarding land application of biosolids, Pepper and colleagues [13] reviewed the literature and concluded that while land application results in temporarily elevated levels of AMR bacteria and ARGs in the soil, significant bacterial die-off occurs relatively quickly. Survival or adaptation of AMR in soil is highly variable and based on numerous site-specific factors, including the presence of pathogens indigenous to the natural soil environment and abiotic and biotic stressors [13,147]. The overall increase in AMR bacteria in soil after biosolids application may be minimal when compared to the composition of indigenous bacteria in soil prior to biosolids application [13]. Based on the available literature, the majority of AMR bacteria and ARGs in soil are likely due to natural phenomena rather than anthropogenic activity [13]. However, there is scant literature on the clinical relevancy of AMR bacteria introduced to the environment from biosolids. Several studies have reported occurrence of antibiotic resistant pathogens including ESBL-producing *E. coli*, MRSA, and vancomycin-resistant *Enterococcus* spp. (VRE) in biosolids [121,150,151]. Across these studies, the detection of pathogens appears to be a particular concern for biosolids that do not undergo advanced treatment such as thermal treatment or lime stabilization. More data are needed on the relative survival of AMR bacteria following land application of biosolids, public health risks associated with indigenous AMR bacteria and ARGs in soil compared to those introduced via applied biosolids, and interactions between introduced and indigenous AMR bacteria and ARGs in soil [13,151]. 

Runoff from rainfall can carry excess contaminants from biosolids to surface waters, including those used for recreation. Proximity to surface water is an important consideration when applying biosolids [145]. More data are needed to determine the contribution of AMR bacteria and ARGs in runoff following land application of biosolids. The concern for HGT is higher in surface water than in soil because aqueous environments allow freer mixing and contact between microorganisms. In soils, separation of cells makes dispersion of genetic elements less likely than in water [13,152]. 

### 3.4. Agriculture and Aquaculture

Antimicrobials are used in agriculture, including animal husbandry and plant crops, and aquaculture to treat and prevent diseases and to promote growth and feed efficiency [153]. Globally, 73% of all antimicrobials sold are used in food animal production [154]. Available estimates indicate that approximately 17.8 million pounds of antimicrobials were used annually in U.S. livestock [153,155]. More data on the global use of antimicrobials in aquaculture are needed, and available data vary widely [153]. Available estimates indicate that approximately 196,000 kg (433,000 pounds) of antibiotics are used annually in U.S. aquaculture alone [156]. Although the U.S. is a leading consumer of fish and fishery products, only 5% to 7% of these products are produced in the U.S., and approximately 90% of U.S. consumed seafood is imported [156]. Therefore, the global use of antimicrobials in aquaculture is likely much larger. In the U.S., it is estimated that the amount of antibiotics used on plants is less than 0.5% of the approximately 22,680,000 kg of antibiotics produced each year [157]. Further, the amount of antibiotics applied in orchards is approximately 0.12% of the amount of total antibiotics used in animal agriculture [158]. 

Animal feeding operations (AFOs) and confined animal feeding operations (CAFOs) are a large source of the antibiotics and it is estimated that between 60 and 80 percent of livestock and poultry routinely receive antimicrobials [153]. The presence of AMR bacteria and ARGs in the environment near AFOs and CAFOs, and the contributions from these sources, have been documented extensively [159,160,161,162,163,164]. Food-animal production in the U.S. utilizes upwards of 25 million pounds of antimicrobials each year [165], and food-animal agriculture produces 1.2 to 1.37 billion tons of animal fecal waste annually [166]. Antibiotics are excreted in animal fecal waste at a rate of approximately 13.5 million pounds annually in the U.S. [159]. These waste products are released into the environment via manure, soil, runoff, or released as wastewater effluent into surface water and sediments [167,168]. At one swine CAFO, elevated downstream concentrations of antibiotic-resistant enterococci were observed when antibiotics were used for non-therapeutic purposes, leaking waste storage pits were present, and manure was land applied [162]. The median concentrations of enterococci detected in downstream surface waters impacted by this CAFO were 17-fold higher than concentrations upstream [162]. Tetracycline resistance was also found to be higher in samples collected downstream than upstream [162]. In a separate study, the abundance and distribution of ARGs in lagoons downstream of cattle CAFOs were found to vary by level of tetracycline use: no use, mixed-use, or high use [161]. Six tetracycline resistance genes were detected in wastewater leaving these CAFOs, and a seasonal pattern was observed with ARGs more frequently detected at high-use facilities in autumn, which is when calves often undergo a 5-day tetracycline dosing program [161]. Runoff and fecal pollution from agricultural and livestock operations also act as sources of ARGs to natural stream sediments [168]. Suttner et al. [168] concluded that inputs of tetracycline resistance genes in stream sediments come from both upstream cattle ranches and natural reservoirs of these ARGs.

The animal supply chain also includes slaughter, rendering, and carcass disposal steps of the animal husbandry process, and bacteria from these sources are known to enter ambient waters. AMR of *Campylobacter jejuni* and *Campylobacter coli* was assessed in a survey of 200 cecal samples of chickens ready for slaughter from 20 major producers in Australia [169]. Of the 108 *C. jejuni* isolates, the most commonly detected resistance was to tetracycline (n = 24) followed by resistance to the quinolones, ciprofloxacin (n = 16) and nalidixic acid (n = 16) [169]. *C. coli* isolates exhibited less overall AMR. However, 5 of the 96 isolates were resistant to ciprofloxacin, nalidixic acid, azithromycin, erythromycin, and clindamycin, respectively, 4 isolates were resistant to telithromycin, and 3 isolates were resistant to tetracycline [169]. Additionally, several studies have found AMR bacteria in untreated wastewater released directly to surface waters from slaughterhouse and animal/meat processing plants, which has led researchers to conclude that these are significant local sources of AMR bacteria [170,171,172,173,174]. In a study of 98 U.S slaughterhouses, 29 meat processing plants exceeded permitted effluent limits for bacteria, including fecal coliforms, *E. coli*, and enterococci, with 119 violations of NPDES permits between January 2016 and June 2018 (note that EPA’s Meat and Poultry products Effluent Guidelines are incorporated into NPDES permits: https://www.epa.gov/eg/meat-and-poultry-products-effluent-guidelines) [175]. The median plant examined for the study had a slaughterhouse wastewater volume similar in magnitude to a small town of 14,000 people and at the high end, equivalent to the load from a city of 79,000 people [175]. During animal disease outbreaks, emergency responses may require largescale carcass disposal and utilization of disinfectants to clean equipment, vehicles, and other potentially contaminated surfaces before movement off site [176]. The disposal of livestock carcasses in the wake of large-scale mortalities may result in the introduction of AMR bacteria and ARGs into the environment via leachate that enters groundwater and runoff into surface waters [177]. The amount of AMR bacteria and ARGs introduced to the environment would vary with the size of the load for disposal. More data are needed to understand the relative importance of this potential pathway. 

Plant crops are potential sources of AMR because antimicrobial pesticides are utilized to disinfect or inhibit growth of unwanted microorganisms on crops and equipment [7,177]. Following application, agricultural runoff can introduce antimicrobial pesticides and AMR bacteria to the environment and affect surface water quality [7,178]. Relative to livestock, less is known about the contribution of plant crops to the amount of antibiotics, AMR bacteria, and ARGs in the environment. The number of studies focused on the contribution of AMR bacteria and ARGs from plant crops is limited primarily to studies of fruit trees and orchards. The available data indicate that AMR bacteria are present at apple orchards regularly treated with streptomycin to prevent *Erwinia amylovora*, the fire blight pathogen, and *Pseudomonas syringae*, the cause of blister spots [179,180,181,182]. For example, a study of apple orchards in New York found streptomycin-resistant bacteria were present in samples of apple leaves (mean = 0.7%) and surrounding soil (mean = 1.5%) [183]. No significant relationships between orchard type or previous streptomycin usage and the percentage of bacteria that were resistant to streptomycin could be found [183]. In a separate study assessing the impact of streptomycin use in commercial apple orchards on bacterial community structure on apple leaves, 43% to 59% (mean = 50%) of the total culturable bacteria were resistant to streptomycin, whereas 57% to 72% (mean = 65%) of the total culturable bacteria were resistant to streptomycin at non-sprayed orchards, illustrating that AMR bacteria are present at apple orchards both treated and not treated with antibiotics [182]. The use of antibiotics appeared affective at reducing bacterial load as a higher number of total culturable bacteria were observed at non-sprayed orchards compared to orchards sprayed with streptomycin [182]. 

Aquaculture (or “aquafarming”) is frequently used in seafood production. In the U.S., there are over 2250 freshwater aquaculture farms and 875 saltwater aquaculture farms equaling over 249,000 and 214,000 surface acres, respectively [184]. Aquaculture systems are generally classified as open, semi-closed, or closed; recreational exposure to water used in both open and semi-open systems is plausible [185]. In open systems, seafood farming occurs in natural waterbodies such as oceans, lakes, rivers, or estuaries [185]. In semi-closed systems, seafood production occurs on land, and water is exchanged between the farm and a natural waterway [185]. 

Antibiotics, other pharmaceuticals, and metal-containing products are used in aquaculture to prevent fouling and to feed and treat fish which leads to selection of antibiotic resistance in the aquatic environment [186]. Up to 80% of antimicrobials administered to fish are excreted in feces and urine in active form [7]. AMR bacteria can also enter aquaculture systems when manure from swine or poultry that have been treated with antimicrobials is used as feed or feed supplement (e.g., pond-raised tilapia) or when runoff water potentially contaminated with human or animal waste contaminates fishponds. AMR bacteria can move from aquaculture systems to surface waters when aquaculture water runs-off, spills, or mixes with surface waters. This can also happen indirectly when sediment from retention aquaculture production ponds (i.e., lagoons) are used as fertilizer in horticulture (e.g., berry production) and subsequently runoff into surface waters [7]. Aquaculture systems have become genetic hotspots for gene transfer in seawater bacteria due to the mixing of feed combinations, which include antibiotics, probiotics, and prebiotics [186,187,188]. A systematic literature review and meta-analysis found that the multi-antibiotic resistance (MAR) index of aquaculture-related bacteria correlates with MAR indices from human clinical bacteria [189]. The review included data from 40 countries, which account for 93% of the global animal aquaculture production. Twenty-eight countries out of the 40 studied displayed MAR indices higher than 0.2, a threshold considered to be an indication of high-risk antibiotic contamination [189].

### 3.5. Birds and Other Wildlife

Seagulls, birds, land mammals, cetaceans, and other wildlife may also be contributors of AMR bacteria and ARGs in the environment [190,191,192,193,194,195,196]. However, the human health risks associated with AMR bacteria and ARGs in wildlife have not been fully evaluated, given the lack of surveillance data for clinically relevant AMR bacteria and ARGs in wildlife [193]. Similarly, it is not clear how much human activities contribute to the occurrence of AMR in wildlife, although one study found that genetic markers of pathogens and ARGs in deer feces were spatially associated with collection near CAFOs (*Campylobacter* spp., *tetQ*, and *ermB*) and land-applied biosolids (*tetQ*) [197]. Birds and mammals may act as both vectors and reservoirs for the spread of AMR, contaminating animal feed, pastures, urban environments, drinking water reservoirs, and recreational waters [194]. For example, Dolejska and Papagiannitsis [194] reviewed studies of ARGs in wildlife species and report ARGs detected from the feces of species, such as wild boar, corvids, American crow, mallards, seagulls, rooks, great cormorant, mouflon, kelp gull, west black-headed gull, African rat, urban rat, hedgehog, and unspecified waterfowl. Additionally, Guenther et al. [198] reviewed studies of ESBL-producing *E. coli* in wildlife fecal samples and report that ESBL-producing *E. coli* has been isolated from a variety of animals, including geese, ducks, deer, fox, owl, gulls, brown and Norway rats, and wild boar. 

The role of wild birds and seagulls in the spread of AMR bacteria and ARGs warrants further evaluation due to their long-distance migration patterns [194]. The feces from wild birds, considered ubiquitous in most areas, are dispersed into the environment where they might contaminate surface waters directly or through surface runoff [193]. Evidence in the literature indicates that seagulls, shorebirds, and geese carry AMR bacteria and ARGs [190,194,198,199]. Additionally, gulls have long been known to carry human pathogens and human source markers (e.g., *Salmonella* spp. and HF183, respectively), with carriage rates associated with proximity to sewage outfalls [192,200]. Seagull feces may contain *E. coli* and enterococci and carry markers of AMR from human waste sites (e.g., sewage outfalls, landfills) to beaches, where they may be further dispersed [192]. 

A study in California identified patterns in antibiotic resistance that aligned with time periods when bird feces were the main source of enterococci contamination in the surf zone [201]. Another study in the Great Lakes identified gulls as the main source of fecal contamination and a high degree of variability for bacteria and resistance, with some samples showing a high-level of resistance to medically relevant antibiotics [202]. Additionally, ESBL-producing *E. coli* were detected in 45 of 139 *E. coli* isolates from seagull feces collected at beaches in Portugal [203]; in 16 of 180 *E. coli* isolates collected from two colonies of yellow-legged gulls in France [204]; in 65 isolates from migratory birds and birds of prey in remote areas of Germany [205]; and 37 isolates from other migratory birds and birds of prey in the Mongolian desert [205]. 

## 4. Health Assessments

### 4.1. Health Endpoints and Pathogens of Concern

A variety of health endpoints are associated with exposure to fecal contamination in ambient waters, including gastrointestinal (GI) illness, urinary tract infections (UTIs), respiratory illnesses, and eye, ear, and skin infections. GI illness has been used as an index health endpoint in many recreational microbial risk assessments because it is often considered the most sensitive and prevalent health endpoint [37,206,207]. Viruses are likely the dominant cause of GI illness associated with recreational water exposures [177]. However, bacterial pathogens such as *S. aureus* can potentially cause more severe health outcomes when drug-resistant strains (i.e., MRSA) are present. MRSA is a growing public health problem, can be fatal, and has been found in ambient recreational waters in the U.S. For example, Plano et al. [18] collected 1001 water samples at a Florida beach and found 248 of 668 water samples collected near bathers and 102 of 333 ambient water samples to be positive for *S. aureus.* A total of 1050 methicillin-sensitive *S. aureus* (MSSA) and 17 MRSA isolates were collected and analyzed from samples collected near bathers; 272 MSSA and 2 MRSA were isolated from ambient water samples [18]. The authors suggest that humans are a potential direct source for *S. aureus* in marine water. Additionally, Thapaliya et al. [15] evaluated the prevalence of MSSA and MRSA at ten freshwater beaches in Northeastern Ohio and found 11.4% of water samples to be positive for MRSA (8 out of 70 samples) with 18.6% of samples positive for MSSA (13 out of 70 samples). The results of these studies indicate that ambient water at certain coastal marine and freshwater beaches in the U.S. may be contaminated with *S. aureus*, including MRSA and MSSA. The authors indicate that further studies are necessary to draw public health conclusions.

### 4.2. Epidemiological Studies

The human health risks related to exposure to AMR bacteria and ARGs in recreational waters have been evaluated using epidemiological methods and risk assessment models. Though limited in number, epidemiological studies have evaluated the association between potential AMR bacteria exposure through recreational water activities and health endpoints such as GI illness [14] and gut colonization by AMR bacteria [17]. Other studies have focused on modeling the probability of human exposure to AMR bacteria in recreational waters by using the prevalence of AMR bacteria measured at various recreational beaches and estimates of water ingestion through swimming [30,208] and various water sports [16,17,28].

In California, Griffith et al. [14] conducted prospective cohort studies at three beaches to examine the association between a variety of bacterial and viral indicators of fecal contamination and the incidence of GI illness. MRSA was strongly associated with GI illness (odds ratio (OR) = 3.49; 95% CI: 1.35–9.06). MRSA was found to be a better predictor of GI illness, as compared to enterococci measured by EPA Method 1600 (a standard culture method) at a beach impacted by human sewage from leaking sewer pipes. This work highlights that MRSA is a strong indicator of health risks from human sewage contamination in recreational waters.

In the United Kingdom, Leonard et al. [17] conducted a cross-sectional epidemiology study comparing surfers who regularly use coastal waters and non-surfers rarely exposed to coastal waters to evaluate the association between coastal water exposure and gut colonization by antibiotic-resistant *E. coli*. Surfers were significantly more likely to be carriers of cefotaxime-resistant *E. coli* (risk ratio (RR) = 2.95; 95% CI: 1.05–8.32) and *bla*_CTX-M_-bearing *E. coli* (RR = 4.09; 95% CI: 1.02–16.4). The *bla*_CTX-M_ genes represent nearly 80% of all ESBL-producing *Enterobacteriaceae*, which confer resistance to multiple antibiotics, such as fluoroquinolones, aminoglycosides, and tetracyclines and are characterized as a serious threat by the CDC’s 2012 and 2019 antibiotic resistance threats reports [1]. While surfers colonized by the AMR bacteria may be asymptomatic, the authors note they may end up more susceptible to infections if they later develop a health condition or pathogenic infection. Additionally, colonized individuals may spread AMR bacteria among the wider population.

Søraas et al. [81] conducted a case–control study in Eastern Norway to evaluate the risk factors for community-acquired UTIs caused by ESBL-producing *Enterobacteriaceae*. Recreational freshwater swimming within the past year was identified as one of several independent risk factors (OR = 2.1; 95% CI: 1.0–4.0). The authors suggest that swimming may be a risk factor for intestinal colonization with *E. coli* with ESBL, and any subsequent UTI may be caused by a newly acquired ESBL-producing strain from the water. While this may highlight a possible connection between environmental pollution and resistant infections, the authors note that the link needs to be further investigated.

### 4.3. Exposure Estimates and Risk Assessments

Few studies have been conducted to estimate human health risks associated with AMR in recreational waters, with preliminary work focusing on exposure assessments. In the Netherlands, Schijven et al. [30] measured concentrations of ESBL-producing *E. coli* in recreational waters and in water in ditches surrounding poultry farms and municipal wastewater. Taking into consideration the effects of dilution and inactivation, the potential of ESBL-producing *E. coli* in source waters to reach downstream recreational waters was modeled, and the probability of human exposure through swimming was estimated. The authors suggest that exposure to ESBL-producing *E. coli* is possible when swimming in recreational waters located downstream of municipal WWTPs or livestock farms. However, the authors also note that the public health risk cannot yet be determined because the exposure is complex and more research is needed, for example, on colonization following asymptomatic carriage and HGT within the gut.

Leonard et al. [28] estimated human exposure to AMR bacteria during recreational water activities conducted in England and Wales. The authors measured the prevalence of ESBL-producing *E. coli* resistant to third generation cephalosporins (3GCs) in coastal water samples and incorporated *E. coli* density data and reported volumes of water ingested during various water sports (e.g., swimming, surfing, diving, boating, wading/splashing). Together, these data provided the mean number of 3GC-resistant ESBL-producing *E. coli* ingested during each water sport. Despite a low prevalence of 3GC-resistance in *E. coli* (0.12%), the authors conclude that there is still an identifiable human exposure risk for recreational water users. Based on their analysis, over 6.3 million water sport sessions in 2012 were estimated to have resulted in the ingestion of at least one 3GC-resistant *E. coli*. Since this study only considered resistance to 3GCs, it likely underestimates the true recreational exposure to AMR bacteria.

In a separate study, Leonard et al. [16] developed a novel targeted metagenomic method to quantify the abundance and diversity of ARGs in *E. coli* and also estimated human exposure to AMR bacteria in recreational water. The authors analyzed sequence data from *E. coli* metagenomes collected from 13 bathing water sites in England to determine the diversity and abundance of associated ARGs. The number of ARGs ingested by recreators was estimated using these sequence data, *E. coli* concentration data collected as part of The Environment Agency’s routine monitoring of 215 English bathing waters (samples collected weekly between mid-May and the end of September), and estimated water ingestion volumes associated with various recreational activities. *E. coli* in these bathing waters were found to harbor, on average, 1.24 ARGs per cell, and it was estimated that recreators ingested at least 100 *E. coli*-borne ARGs during each recreational water activity session [16].

O’Flaherty et al. [208] examined potential human exposures to antibiotic resistant *E. coli* in two rivers located near different WWTPs and recreational beaches in central Italy. The authors conducted a field survey to better understand how much water was consumed by beachgoers and created a quantitative risk assessment model using dilution and decay rates of antibiotic resistant *E. coli* from the WWTP effluent into the river, dilution rates from the river into the swimming areas, and incidental water ingestion rates from swimming. The mean predicted human exposure levels (calculated by multiplying the predicted quantity of contamination at the bathing water site by the quantity of water consumed during recreation) to antibiotic resistant *E. coli* were between 0 and 345 colony-forming units per 100 milliliters. Such modeling suggests that human exposure to resistance occurs through recreational water activities.

## 5. Future Research Needs

This paper summarized available information on possible sources of AMR bacteria and ARGs in ambient surface waters and human recreational exposures to AMR bacteria and ARGs using available human health studies. Regarding our understanding of recreational water exposures and AMR risks, this review exposed a number of knowledge gaps and research challenges including the following: (1) information on contributions on various sources to environmental AMR; (2) behavior of AMR bacteria and ARGs in the environment (specifically in ambient water and across wastewater treatment processes); (3) human health risks associated with recreational exposure to AMR bacteria and ARGs in water; and (4) standardized methods for culturable and gene targets. 

### 5.1. Source Contribution

Additional data on environmental sources of AMR bacteria and ARGs and their associated contributions are needed. Amarasiri et al. [33] provided occurrence information on AMR bacteria, ARGs, and antibiotics in various water types, including fresh and marine waters used for recreation, but data collected in the U.S. are currently limited. In addition, the many sources of AMR bacteria and ARGs (e.g., medical waste, municipal wastewater, agricultural applications) illustrate how AMR bacteria and ARGs can easily spread to the environment, but their relative contributions to recreational waters are unknown [33]. Other common environmental stressors and sources of human fecal contamination might also introduce AMR bacteria and ARGs to the environment. Contributions from landfills, septic tanks, cesspools, and cemeteries can leach through soil directly into groundwater seeping into the recreational water environment [209,210]. Swimmers and bathers in recreational waters might also serve as a source of AMR bacteria and ARGs as they can shed fecal material while recreating [211]. Published evidence linking these sources to AMR bacteria and ARGs in recreational water is not available.

A better characterization of potential sources and their respective contributions would help with quantifying exposure, risk assessment modeling and possible mitigation strategies. Specifically, more data are needed on the variability and load contributions of potential key sources of AMR bacteria and ARGs (i.e., WWTPs, AFOs/CAFOs, medical/healthcare facilities, and wildlife) in surface waters. For medical/health care facilities, a better understanding of their impact on municipal wastewater systems would inform future best practices and treatment needs. Active surveillance in surface waters of clinically relevant AMR bacteria strains is needed, as environmental surveillance could provide added protection of human, animal, and ecosystem health [212]. Huijbers et al. [212] proposed a framework for environmental surveillance and direct assessment of sites, where human exposure is likely to occur as the most informative for assessing the risk of transmission of AMR bacteria and genes. Exposure-relevant sites include recreational areas (beach sand and swimming water), drinking water, and sewage. In addition to AFO/CAFOs, there is a potential for AMR bacteria and ARGs to enter surface waters from slaughterhouse waste streams [175]. Studies evaluating the impact of slaughterhouse waste on surface waters from an AMR perspective are needed.

### 5.2. Fate and Transport—Across Treatment and in the Environment

Despite the environmental and public health significance of the well-known fecal contamination sources, there are data gaps regarding how AMR from these sources persist in the environment, across wastewater treatments, and what concentrations may pose a human health risk. A better understanding of how AMR bacteria and ARGs persist in water and wastewater would help WWTPs develop a more targeted approach to removing AMR bacteria and ARGs, thereby reducing their presence in effluent discharged into surface waters. Treatment processes are complex, and the effectiveness of disinfectants may be affected by a variety of factors such as dose, contact time, temperature, and pH [130,131]. Another important consideration is the potential selection for AMR bacteria in wastewater treatment systems [213].

While the presence of AMR bacteria and ARGs in ambient surface water has been well-documented, their behavior in the environment and response to selection pressure is unclear [31,33,214]. Pepper et al. [13] describe a need to better understand the survival of AMR bacteria and the transfer efficiency of ARGs in various environmental matrices including water and soil. In particular, there is limited information on how pathogenic AMR bacteria survive in the natural environment compared to non-AMR bacteria, the endemic levels of AMR bacteria and ARGs in pristine soils, and how introduced and indigenous AMR bacteria and ARGs may interact [13]. To address this data gap, Amarasiri et al. [33] propose adopting the assumption that pathogenic AMR bacteria will display similar behavior in the environment as nonpathogenic AMR bacteria, but more research is needed to evaluate the validity of this assumption.

### 5.3. Human Health Risks

The existing literature lacks a well-developed approach for the development of human health risk assessments of AMR bacteria and ARG exposure in recreational water. Risk assessments of how AMR bacteria and ARGs interact with environmental bacteria and threaten human populations are lacking [32,33,85]. As described by Schijven et al. [30], a quantitative microbial risk assessment (QMRA) can evaluate the human health risk from AMR. However, there is a need for more data on exposure pathways, and dose–response relationships must be derived for various health outcomes (e.g., difficult-to-treat infection or carriage of resistant commensal bacteria with the risk of horizontal transfer of resistance genes to pathogens) before the risk assessments are truly quantitative. The studies by Schijven et al. [30], Leonard et al. [28], and O’Flaherty et al. [208] all estimated the likelihood of human exposure to AMR bacteria in recreational waters, which is an important first step in QMRA. Ben et al. [32] proposed a conceptual framework of human health risk to facilitate conducting QMRAs of antibiotic resistance associated with antibiotic residues in the environment. After conducting an intensive review of the available literature on antibiotic residues and AMR bacteria and ARGs in the environment (including drinking water, dust, soil, meat, milk, edible crop/vegetable, aquatic products, surface water, sediment, manure, air, raw sewage, and WWTP effluent), Ben and colleagues concluded that the data are insufficient to use the QMRA conceptual framework [32]. Important data gaps include the paucity of data on human exposure to AMR bacteria and ARGs in the environment, which could be remedied with monitoring data to quantify residual levels of antibiotics, AMR bacteria, and ARGs during environmental exposures (e.g., recreational surface waters exposure). Risk assessments could also consider important environmental factors, such as co-occurrence of heavy metals and the selective pressures heavy metals have on ARGs [70]. Given the data gaps in QMRAs, Ashbolt et al. [215] suggests using a multicriteria decision analysis (MCDA) approach which provides a structured framework for making choices and ranking risks when multiple factors need to be considered. For example, MCDA approaches could be used to evaluate and rank the relative risks between relevant contributing factors such as the mobility of resistance determinants in genetic elements, antibiotic resistance transfer rates in different environmental compartments, accumulation levels of antibiotics in environmental compartments, and environmental fate and transport to exposure points [215]. These MCDA approaches could result in tools to better assess the most effective control points for reducing health risks and preventing the further development of AMR [215].

Additionally, before a quantitative risk assessment can be conducted, there is a need for formal dose–response models with respect to specific pathogenic AMR bacteria [13,30,32,33]. Because bacterial responses to antibiotics are concentration-dependent, a dose–response assessment of the relationship between the evolution and emergence of antibiotic resistance and antibiotic concentrations requires a metric to indicate the potential of antibiotic concentrations to promote the development of AMR bacteria in complex bacterial communities. Existing studies have focused primarily on the likelihood of exposure and have not established levels of harm or relevant health outcomes. In addition to ingestion, alternate routes of exposure should be considered, such as dermal and inhalation [13]. Aside from GI illness, other relevant health outcomes may include respiratory, skin, eye, ear, or urinary tract infections, or perhaps gut colonization, as it can lead to subsequent resistant infections [17]. Santiago-Rodriguez et al. [25] expressed concern that AMR bacteria and ARGs involved in skin infections are present in recreational waters; however, it has yet to be determined what kind of health risks they may pose, beyond MRSA.

### 5.4. Standardized Methods

No standard methods exist for measuring AMR in recreational waters. A general observation of the available literature is that researchers are using a variety of different methods to study environmental AMR with variations in the bacteria or genes targeted and the antibiotics tested, which precludes cross comparison of datasets. To develop a standardized method, researchers will likely need to decide whether to adopt culture-based methods, molecular methods, or both. For the molecular methods, there will also need to be consensus on the appropriate gene targets. Several gene targets appear promising as indicators of environmental AMR, including *int*I [216,217], *mcr-1* [218,219], *sul*1 [220,221], *tet*W [220,221], *bla*_TEM_ [222,223], and others [115,224,225]. A future systematic review and inventory of gene targets could prove useful for helping prioritize the development and standardization of molecular methods. Improving technologies for PCR arrays are also making it more feasible to simultaneously test for multiple AMR gene targets within a single assay [226]. Some researchers are also using sequencing approaches. Metagenomic approaches can be used to catalogue occurrence of antibiotic resistance genes in environmental samples (e.g., Li et al. [225]), or a combination of quantitative culture with whole genome sequencing can be used to determine what genes are carried by a bacterial species of interest (e.g., Leonard et al. [17]) and to predict phenotypic resistance (e.g., McDermott et al. [227]). The cost of sequencing and metagenomic approaches may be prohibitive for routine water monitoring but could become a more viable option as methods improve and costs decrease in the future.

Regarding a culturable method for targeting AMR in recreational waters, *E. coli* may be useful as it is a standard water quality indicator and its evaluation would complement existing efforts for surveillance of AMR. In the U.S., resistant *E. coli* is monitored by all three federal agencies (CDC, FDA, and USDA) collaborating in the National Antimicrobial Resistance Monitoring System (NARMS) aimed at monitoring trends in AMR from humans, animals, and retail meats [228]. Additionally, the WHO integrated global survey protocol, developed through the Tricycle Project, has selected *E. coli*, specifically ESBL-*E. coli*, for global surveillance spanning human, animal, and environmental samples [229,230,231]. Use of a similar, culture-based assay for ESBL*-E. coli* in recreational waters would ensure testing of viable organisms with human health relevance. Use of culture-based *E. coli* would also facilitate comparison of data for recreational waters and other types of samples across national and international databases.

Addressing these research needs will help inform policies and practices to reduce the spread of AMR into the environment and protect recreational water users from exposure.

## 6. Conclusions

As microbial resistance to antibiotics and multidrug resistant strains rise within the natural environment and clinical settings, improving antibiotic stewardship has become more pressing [1]. When evaluating ways to protect human health during recreational activities in surface waters, AMR bacteria may be an important consideration. However, at this point in time, data on both natural reservoirs and the ability of treatment processes to remove antibiotics, AMR bacteria and ARGs are fragmented. Additionally, more data are needed to determine the volumes and concentrations of antibiotics, active pharmaceutical ingredients, and AMR bacteria and ARGs being discharged into surface waters used for recreation. Surface water monitoring and/or targeted surveillance data on the prevalence, concentration, and location of AMR bacteria are key to begin developing human health risk assessments and prioritizing the AMR risks to human populations. 

## References

[1] Centers for Disease Control and Prevention (CDC) (2019). Antibiotic Resistance Threats in the United States.

[2] The Council of Canadian Academies (2019). When Antibiotics Fail: The Expert Panel on the Potential Socio-Economic Impacts of Antimicrobial Resistance in Canada.

[3] World Health Organization (WHO) (2019). Ten Threats to Global Health in 2019.

[4] Aslam B., Wang W., Arshad M.I., Khurshid M., Muzammil S., Rasool M.H., Nisar M.A., Alvi R.F., Aslam M.A., Qamar M.U. (2018). Antibiotic resistance: A rundown of a global crisis. Infect. Drug Resist..

[5] Robinson T.P., Bu D.P., Carrique-Mas J., Fèvre E.M., Gilbert M., Grace D., Hay S.I., Jiwakanon J., Kakkar M., Kariuki S. (2016). Antibiotic resistance is the quintessential One Health issue. Trans. R. Soc. Trop. Med. Hyg..

[6] World Health Organization (WHO) (2019). Antimicrobial Resistance.

[7] Food and Agricultural Organization (FAO), World Health Organization (WHO) (2019). Joint FAO/WHO Expert Meeting in Collaboration with OIE on Foodborne Antimicrobial Resistance: Role of the Environment, Crops and Biocides.

[8] World Health Organization (WHO) (2020). Technical Brief on Water, Sanitation, Hygiene and Wastewater Management to Prevent Infections and Reduce the Spread of Antimicrobial Resistance.

[9] Martinez J.L. (2009). The role of natural environments in the evolution of resistance traits in pathogenic bacteria. Proc. Biol. Sci..

[10] Wright G.D. (2010). Antibiotic resistance in the environment: A link to the clinic?. Curr. Opin. Microbiol..

[11] Williams-Nguyen J., Sallach J.B., Bartelt-Hunt S., Boxall A.B., Durso L.M., McLain J.E., Singer R.S., Snow D.D., Zilles J.L. (2016). Antibiotics and antibiotic resistance in agroecosystems: State of the science. J. Environ. Qual..

[12] Quintela-Baluja M., Chan C.W., Ezzat Alnakip M., Abouelnaga M., Graham D.W. (2015). Sanitation, water quality and antibiotic resistance dissemination. The Battle against Microbial Pathogens: Basic Science, Technological Advances and Educational Programs.

[13] Pepper I.L., Brooks J.P., Gerba C.P. (2018). Antibiotic resistant bacteria in municipal wastes: Is there reason for concern?. Environ. Sci. Technol..

[14] Griffith J.F., Weisberg S.B., Arnold B.F., Cao Y., Schiff K.C., Colford J.M. (2016). Epidemiologic evaluation of multiple alternate microbial water quality monitoring indicators at three California beaches. Water Res..

[15] Thapaliya D., Hellwig E.J., Kadariya J., Grenier D., Jefferson A.J., Dalman M., Kennedy K., DiPerna M., Orihill A., Taha M. (2017). Prevalence and characterization of Staphylococcus aureus and methicillin-resistant Staphylococcus aureus on public recreational beaches in Northeast Ohio. Geohealth.

[16] Leonard A.F.C., Yin X.L., Zhang T., Hui M., Gaze W.H. (2018). A coliform-targeted metagenomic method facilitating human exposure estimates to Escherichia coli-borne antibiotic resistance genes. FEMS Microbiol. Ecol..

[17] Leonard A.F.C., Zhang L., Balfour A.J., Garside R., Hawkey P.M., Murray A.K., Ukoumunne O.C., Gaze W.H. (2018). Exposure to and colonisation by antibiotic-resistant E. coli in UK coastal water users: Environmental surveillance, exposure assessment, and epidemiological study (Beach Bum Survey). Environ. Int..

[18] Plano L.R., Shibata T., Garza A.C., Kish J., Fleisher J.M., Sinigalliano C.D., Gidley M.L., Withum K., Elmir S.M., Hower S. (2013). Human-associated methicillin-resistant Staphylococcus aureus from a subtropical recreational marine beach. Microb. Ecol..

[19] Dekker J.P., Frank K.M. (2015). Salmonella, Shigella, and Yersinia. Clin. Lab. Med..

[20] Fogarty L.R., Haack S.K., Johnson H.E., Brennan A.K., Isaacs N.M., Spencer C. (2015). Staphylococcus aureus and methicillin-resistant S. aureus (MRSA) at ambient freshwater beaches. J. Water Health.

[21] Sokari T.G., Ibiebele D.D., Ottih R.M. (1988). Antibiotic resistance among coliforms and Pseudomonas spp. from bodies of water around Port Harcourt, Nigeria. J. Appl. Bacteriol..

[22] Ghosh A.R., Nair G.B., Naik T.N., Sarkar S.K., Mazumdar R., Pal S.C., Sen D. (1991). Serovars of multi-antibiotic resistant Escherichia coli from the freshwater environs of Calcutta, India. Microbiol. Immunol..

[23] Schwartz T., Kohnen W., Jansen B., Obst U. (2003). Detection of antibiotic-resistant bacteria and their resistance genes in wastewater, surface water, and drinking water biofilms. FEMS Microbiol. Ecol..

[24] Czekalski N., Berthold T., Caucci S., Egli A., Bürgmann H. (2012). Increased levels of multiresistant bacteria and resistance genes after wastewater treatment and their dissemination into Lake Geneva, Switzerland. Front. Microbiol..

[25] Santiago-Rodriguez T.M., Rivera J.I., Coradin M., Toranzos G.A. (2013). Antibiotic-resistance and virulence genes in Enterococcus isolated from tropical recreational waters. J. Water Health.

[26] Colomer-Lluch M., Mora A., Lopez C., Mamani R., Dahbi G., Marzoa J., Herrera A., Viso S., Blanco J.E., Blanco M. (2013). Detection of quinolone-resistant Escherichia coli isolates belonging to clonal groups O25b:H4-B2-ST131 and O25b:H4-D-ST69 in raw sewage and river water in Barcelona, Spain. J. Antimicrob. Chemother..

[27] Alm E.W., Zimbler D., Callahan E., Plomaritis E. (2014). Patterns and persistence of antibiotic resistance in faecal indicator bacteria from freshwater recreational beaches. J. Appl. Microbiol..

[28] Leonard A.F., Zhang L., Balfour A.J., Garside R., Gaze W.H. (2015). Human recreational exposure to antibiotic resistant bacteria in coastal bathing waters. Environ. Int..

[29] Overbey K.N., Hatcher S.M., Stewart J.R. (2015). Water quality and antibiotic resistance at beaches of the Galápagos Islands. Front. Environ. Sci..

[30] Schijven J.F., Blaak H., Schets F.M., de Roda Husman A.M. (2015). Fate of extended-spectrum beta-cactamase-producing Escherichia coli from faecal sources in surface water and probability of human exposure through swimming. Environ. Sci. Technol..

[31] Kadykalo S., Thomas J., Parmley E.J., Pintar K., Fleury M. (2020). Antimicrobial resistance of Salmonella and generic Escherichia coli isolated from surface water samples used for recreation and a source of drinking water in southwestern Ontario, Canada. Zoonoses Public Health.

[32] Ben Y., Fu C., Hu M., Liu L., Wong M.H., Zheng C. (2019). Human health risk assessment of antibiotic resistance associated with antibiotic residues in the environment: A review. Environ. Res..

[33] Amarasiri M., Sano D., Suzuki S. (2019). Understanding human health risks caused by antibiotic resistant bacteria (ARB) and antibiotic resistance genes (ARG) in water environments: Current knowledge and questions to be answered. Crit. Rev. Environ. Sci. Technol..

[34] Boczek L.A., Rice E.W., Johnston B., Johnson J.R. (2007). Occurrence of antibiotic-resistant uropathogenic Escherichia coli clonal group A in wastewater effluents. Appl. Environ. Microbiol..

[35] Chang H.H., Cohen T., Grad Y.H., Hanage W.P., O’Brien T.F., Lipsitch M. (2015). Origin and proliferation of multiple-drug resistance in bacterial pathogens. Microbiol. Mol. Biol. Rev..

[36] Bueno I., Williams-Nguyen J., Hwang H., Sargeant J.M., Nault A.J., Singer R.S. (2018). Systematic review: Impact of point sources on antibiotic-resistant bacteria in the natural environment. Zoonoses Public Health.

[37] U.S. Environmental Protection Agency (USEPA) (2012). Recreational Water Quality Criteria.

[38] Health Canada (2012). Guidelines for Canadian Recreational Water Quality.

[39] European Environment Agency (EEA) (2005). Bathing Water Quality.

[40] Rizzo L., Manaia C., Merlin C., Schwartz T., Dagot C., Ploy M.C., Michael I., Fatta-Kassinos D. (2013). Urban wastewater treatment plants as hotspots for antibiotic resistant bacteria and genes spread into the environment: A review. Sci. Total Environ..

[41] Mao D., Yu S., Rysz M., Luo Y., Yang F., Li F., Hou J., Mu Q., Alvarez P.J. (2015). Prevalence and proliferation of antibiotic resistance genes in two municipal wastewater treatment plants. Water Res..

[42] Gantzer C., Maul A., Audic J.M., Schwartzbrod L. (1998). Detection of infectious enteroviruses, enterovirus genomes, somatic coliphages, and Bacteroides fragilis phages in treated wastewater. Appl. Environ. Microbiol..

[43] Debroas D., Siguret C. (2019). Viruses as key reservoirs of antibiotic resistance genes in the environment. ISME J..

[44] Baltz R.H. (2010). Genomics and the ancient origins of the daptomycin biosynthetic gene cluster. J. Antibiot..

[45] Adu-Oppong B., Gasparrini A.J., Dantas G. (2017). Genomic and functional techniques to mine the microbiome for novel antimicrobials and antimicrobial resistance genes. Ann. N. Y. Acad. Sci..

[46] D’Costa V.M., King C.E., Kalan L., Morar M., Sung W.W., Schwarz C., Froese D., Zazula G., Calmels F., Debruyne R. (2011). Antibiotic resistance is ancient. Nature.

[47] Cytryn E. (2013). The soil resistome: The Anthropogenic, the nature and the unknown. Soil Biol. Biochem..

[48] Diaz K.S., Rich V.I., McLain J.E. (2017). Searching for antibiotic resistance genes in a pristine Arctic wetland. J. Contemp. Water Res. Educ..

[49] Durso L.M., Wedin D.A., Gilley J.E., Miller D.N., Marx D.B. (2016). Assessment of selected antibiotic resistances in ungrazed native Nebraska prairie soils. J. Environ. Qual..

[50] Rahman M.H., Nonaka L., Tago R., Suzuki S. (2008). Occurrence of two genotypes of tetracycline (TC) resistance gene tet(M) in the TC-resistant bacteria in marine sediments of Japan. Environ. Sci. Technol..

[51] Taylor E.L.S., Ferreira G.F., de Freitas G.J.C., Ferreira R.L., Assiss D., Santos D., de Resende-Stoianoff M.A. (2017). Screening of antifungal susceptibility in cave dwelling aspergilli and report of an amphotericin B-resistant Aspergillus flavus. Int. J. Speleol..

[52] Bhullar K., Waglechner N., Pawlowski A., Koteva K., Banks E.D., Johnston M.D., Barton H.A., Wright G.D. (2012). Antibiotic resistance is prevalent in an isolated cave microbiome. PLoS ONE.

[53] Kummerer K. (2009). Antibiotics in the aquatic environment—A review—Part I. Chemosphere.

[54] Zhu H., Sandiford S.K., van Wezel G.P. (2014). Triggers and cues that activate antibiotic production by actinomycetes. J. Ind. Microbiol. Biotechnol..

[55] Burmeister A.R. (2015). Horizontal gene transfer. Evol. Med. Public Health.

[56] Davies J., Davies D. (2010). Origins and evolution of antibiotic resistance. Microbiol. Mol. Biol. Rev..

[57] Ventola C.L. (2015). The antibiotic resistance crisis: Part 1: Causes and threats. Pharm. Ther..

[58] Woegerbauer M., Bellanger X., Merlin C. (2020). Cell-Free DNA: An underestimated source of antibiotic resistance gene dissemination at the interface between human activities and downstream environments in the context of wastewater reuse. Front. Microbiol..

[59] Smalla K., Cook K., Djordjevic S.P., Klumper U., Gillings M. (2018). Environmental dimensions of antibiotic resistance: Assessment of basic science gaps. FEMS Microbiol. Ecol..

[60] Joseph S.M., Battaglia T., Maritz J.M., Carlton J.M., Blaser M.J. (2019). Longitudinal comparison of bacterial diversity and antibiotic resistance genes in New York City sewage. MSystems.

[61] Abe K., Nomura N., Suzuki S. (2020). Biofilms: Hot spots of horizontal gene transfer (HGT) in aquatic environments, with a focus on a new HGT mechanism. FEMS Microbiol. Ecol..

[62] Melnyk A.H., Wong A., Kassen R. (2015). The fitness costs of antibiotic resistance mutations. Evol. Appl..

[63] Baker-Austin C., Wright M.S., Stepanauskas R., McArthur J.V. (2006). Co-selection of antibiotic and metal resistance. Trends Microbiol..

[64] Seiler C., Berendonk T.U. (2012). Heavy metal driven co-selection of antibiotic resistance in soil and water bodies impacted by agriculture and aquaculture. Front. Microbiol..

[65] Wellington E.M.H., Boxall A.B.A., Cross P., Feil E.J., Gaze W.H., Hawkey P.M., Johnson-Rollings A.S., Jones D.L., Lee N.M., Otten W. (2013). The role of the natural environment in the emergence of antibiotic resistance in Gram-negative bacteria. Lancet Infect. Dis..

[66] Baquero F., Coque T.M. (2014). Widening the spaces of selection: Evolution along sublethal antimicrobial gradients. MBio.

[67] Gullberg E., Albrecht L.M., Karlsson C., Sandegren L., Andersson D.I. (2014). Selection of a multidrug resistance plasmid by sublethal levels of antibiotics and heavy metals. MBio.

[68] Pal C., Bengtsson-Palme J., Kristiansson E., Larsson D.G. (2015). Co-occurrence of resistance genes to antibiotics, biocides and metals reveals novel insights into their co-selection potential. BMC Genom..

[69] Yang Q.E., Agouri S.R., Tyrrell J.M., Walsh T.R. (2018). Heavy metal resistance genes are associated with blaNDM-1- and blaCTX-M-15-carrying Enterobacteriaceae. Antimicrob. Agents Chemother..

[70] Chen J., Li J., Zhang H., Shi W., Liu Y. (2019). Bacterial heavy-metal and antibiotic resistance genes in a copper tailing dam area in Northern China. Front. Microbiol..

[71] Dickinson A.W., Power A., Hansen M.G., Brandt K.K., Piliposian G., Appleby P., O’Neill P.A., Jones R.T., Sierocinski P., Koskella B. (2019). Heavy metal pollution and co-selection for antibiotic resistance: A microbial palaeontology approach. Environ. Int..

[72] Hiller C.X., Hubner U., Fajnorova S., Schwartz T., Drewes J.E. (2019). Antibiotic microbial resistance (AMR) removal efficiencies by conventional and advanced wastewater treatment processes: A review. Sci. Total Environ..

[73] Almakki A., Jumas-Bilak E., Marchandin H., Licznar-Fajardo P. (2019). Antibiotic resistance in urban runoff. Sci. Total Environ..

[74] Gordon L., Giraud E., Ganiere J.P., Armand F., Bouju-Albert A., de la Cotte N., Mangion C., Le Bris H. (2007). Antimicrobial resistance survey in a river receiving effluents from freshwater fish farms. J. Appl. Microbiol..

[75] Berendonk T.U., Manaia C.M., Merlin C., Fatta-Kassinos D., Cytryn E., Walsh F., Burgmann H., Sorum H., Norstrom M., Pons M.N. (2015). Tackling antibiotic resistance: The environmental framework. Nat. Rev. Microbiol..

[76] Olaniran A.O., Nzimande S.B., Mkize N.G. (2015). Antimicrobial resistance and virulence signatures of Listeria and Aeromonas species recovered from treated wastewater effluent and receiving surface water in Durban, South Africa. BMC Microbiol..

[77] Chaix G., Roger F., Berthe T., Lamy B., Jumas-Bilak E., Lafite R., Forget-Leray J., Petit F. (2017). Distinct aeromonas populations in water column and associated with copepods from estuarine environment (Seine, France). Front. Microbiol..

[78] Ferreira da Silva M., Vaz-Moreira I., Gonzalez-Pajuelo M., Nunes O.C., Manaia C.M. (2007). Antimicrobial resistance patterns in Enterobacteriaceae isolated from an urban wastewater treatment plant. FEMS Microbiol. Ecol..

[79] Galvin S., Boyle F., Hickey P., Vellinga A., Morris D., Cormican M. (2010). Enumeration and characterization of antimicrobial-resistant Escherichia coli bacteria in effluent from municipal, hospital, and secondary treatment facility sources. Appl. Environ. Microbiol..

[80] Łuczkiewicz A., Jankowska K., Fudala-Książek S., Olańczuk-Neyman K. (2010). Antimicrobial resistance of fecal indicators in municipal wastewater treatment plant. Water Res..

[81] Soraas A., Sundsfjord A., Sandven I., Brunborg C., Jenum P.A. (2013). Risk factors for community-acquired urinary tract infections caused by ESBL-producing enterobacteriaceae—A case-control study in a low prevalence country. PLoS ONE.

[82] Hooban B., Joyce A., Fitzhenry K., Chique C., Morris D. (2020). The role of the natural aquatic environment in the dissemination of extended spectrum beta-lactamase and carbapenemase encoding genes: A scoping review. Water Res..

[83] Eftim S.E., Hong T., Soller J., Boehm A., Warren I., Ichida A., Nappier S.P. (2017). Occurrence of norovirus in raw sewage—A systematic literature review and meta-analysis. Water Res..

[84] Soller J.A., Eftim S.E., Nappier S.P. (2018). Direct potable reuse microbial risk assessment methodology: Sensitivity analysis and application to state log credit allocations. Water Res..

[85] Jury K.L., Khan S.J., Vancov T., Stuetz R.M., Ashbolt N.J. (2011). Are sewage treatment plants promoting antibiotic resistance?. Crit. Rev. Environ. Sci. Technol..

[86] U.S. Environmental Protection Agency (USEPA) (2004). 40 CFR Part 431 Effluent Limitations Guidelines and New Source Performance Standards for the Meat and Poultry Products Point Source Category.

[87] U.S. Environmental Protection Agency (USEPA) (2011). Keeping Raw Sewage & Contaminated Stormwater Out of the Public’s Water.

[88] U.S. Environmental Protection Agency (USEPA) (2019). Sanitary Sewer Overflows.

[89] Pouillot R., Van Doren J.M., Woods J., Plante D., Smith M., Goblick G., Roberts C., Locas A., Hajen W., Stobo J. (2015). Meta-analysis of the reduction of norovirus and male-specific coliphage concentrations in wastewater treatment plants. Appl. Environ. Microbiol..

[90] Honda R., Tachi C., Yasuda K., Hirata T., Noguchi M., Hara-Yamamura H., Yamamoto-Ikemoto R., Watanabe T. (2020). Estimated discharge of antibiotic-resistant bacteria from combined sewer overflows of urban sewage system. NPJ Clean Water.

[91] Hua J., An P., Winter J., Gallert C. (2003). Elimination of COD, microorganisms and pharmaceuticals from sewage by trickling through sandy soil below leaking sewers. Water Res..

[92] Paul M., Wolf L., Fund K., Held I., Winter J., Eiswirth† M., Gallert C., Hötzl H. (2004). Microbiological condition of urban groundwater in the vicinity of leaky sewer systems. Acta Hydrochim. Hydrobiol..

[93] Rosi-Marshall E.J., Kelly J.J. (2015). Antibiotic stewardship should consider environmental fate of antibiotics. Environ. Sci. Technol..

[94] Gallert C., Fund K., Winter J. (2005). Antibiotic resistance of bacteria in raw and biologically treated sewage and in groundwater below leaking sewers. Appl. Microbiol. Biotechnol..

[95] Sigala J., Unc A. (2012). A PCR-DGGE approach to evaluate the impact of wastewater source on the antibiotic resistance diversity in treated wastewater effluent. Water Sci. Technol..

[96] Li B., Yang Y., Ma L., Ju F., Guo F., Tiedje J.M., Zhang T. (2015). Metagenomic and network analysis reveal wide distribution and co-occurrence of environmental antibiotic resistance genes. ISME J..

[97] Divyashree M., Mani M.K., Shama Prakash K., Vijaya Kumar D., Veena Shetty A., Shetty A.K., Karunasagar I. (2020). Hospital wastewater treatment reduces NDM-positive bacteria being discharged into water bodies. Water Environ. Res..

[98] Hendriksen R.S., Munk P., Njage P., van Bunnik B., McNally L., Lukjancenko O., Röder T., Nieuwenhuijse D., Pedersen S.K., Kjeldgaard J. (2019). Global monitoring of antimicrobial resistance based on metagenomics analyses of urban sewage. Nat. Commun..

[99] Parnanen K.M.M., Narciso-da-Rocha C., Kneis D., Berendonk T.U., Cacace D., Do T.T., Elpers C., Fatta-Kassinos D., Henriques I., Jaeger T. (2019). Antibiotic resistance in European wastewater treatment plants mirrors the pattern of clinical antibiotic resistance prevalence. Sci. Adv..

[100] U.S. Environmental Protection Agency (USEPA) (2018). Five-Year Review of the 2012 Recreational Water Quality Criteria.

[101] Reinthaler F.F., Posch J., Feierl G., Wust G., Haas D., Ruckenbauer G., Mascher F., Marth E. (2003). Antibiotic resistance of E. coli in sewage and sludge. Water Res..

[102] Pauwels B., Verstraete W. (2006). The treatment of hospital wastewater: An appraisal. J. Water Health.

[103] Lamba M., Graham D.W., Ahammad S.Z. (2017). Hospital wastewater releases of carbapenem-resistance pathogens and genes in urban India. Environ. Sci. Technol..

[104] Levy S.B. (2001). Antibacterial household products: Cause for concern. Emerg. Infect. Dis..

[105] Aiello A.E., Larson E. (2003). Antibacterial cleaning and hygiene products as an emerging risk factor for antibiotic resistance in the community. Lancet Infect. Dis..

[106] Aiello A.E., Marshall B., Levy S.B., Della-Latta P., Lin S.X., Larson E. (2005). Antibacterial cleaning products and drug resistance. Emerg. Infect. Dis..

[107] Hutinel M., Huijbers P.M.C., Fick J., Ahren C., Larsson D.G.J., Flach C.F. (2019). Population-level surveillance of antibiotic resistance in Escherichia coli through sewage analysis. Eurosurveillance.

[108] McArdell C.S., Molnar E., Suter M.J.F., Giger W. (2003). Occurrence and fate of macrolide antibiotics in wastewater treatment plants and in the Glatt Valley Watershed, Switzerland. Environ. Sci. Technol..

[109] Voigt A.M., Zacharias N., Timm C., Wasser F., Sib E., Skutlarek D., Parcina M., Schmithausen R.M., Schwartz T., Hembach N. (2019). Association between antibiotic residues, antibiotic resistant bacteria and antibiotic resistance genes in anthropogenic wastewater—An evaluation of clinical influences. Chemosphere.

[110] Scott T.-M., Phillips P.J., Kolpin D.W., Colella K.M., Furlong E.T., Foreman W.T., Gray J.L. (2018). Pharmaceutical manufacturing facility discharges can substantially increase the pharmaceutical load to U.S. wastewaters. Sci. Total Environ..

[111] Li D., Yang M., Hu J., Ren L., Zhang Y., Li K. (2008). Determination and fate of oxytetracycline and related compounds in oxytetracycline production wastewater and the receiving river. Environ. Toxicol. Chem..

[112] Larsson D.G., de Pedro C., Paxeus N. (2007). Effluent from drug manufactures contains extremely high levels of pharmaceuticals. J. Hazard. Mater..

[113] LaPara T.M., Burch T.R., McNamara P.J., Tan D.T., Yan M., Eichmiller J.J. (2011). Tertiary-treated municipal wastewater is a significant point source of antibiotic resistance genes into Duluth-Superior Harbor. Environ. Sci. Technol..

[114] Czekalski N., Gascón Díez E., Bürgmann H. (2014). Wastewater as a point source of antibiotic-resistance genes in the sediment of a freshwater lake. ISME J..

[115] Cacace D., Fatta-Kassinos D., Manaia C.M., Cytryn E., Kreuzinger N., Rizzo L., Karaolia P., Schwartz T., Alexander J., Merlin C. (2019). Antibiotic resistance genes in treated wastewater and in the receiving water bodies: A pan-European survey of urban settings. Water Res..

[116] Silva J., Castillo G., Callejas L., López H., Olmos J. (2006). Frequency of Transferable Multiple Antibiotic Resistance Amongst Coliform Bacteria Isolated from a Treated Sewage Effluent in Antofagasta.

[117] Korzeniewska E., Korzeniewska A., Harnisz M. (2013). Antibiotic resistant Escherichia coli in hospital and municipal sewage and their emission to the environment. Ecotoxicol. Environ. Saf..

[118] Amador P.P., Fernandes R.M., Prudencio M.C., Barreto M.P., Duarte I.M. (2015). Antibiotic resistance in wastewater: Occurrence and fate of Enterobacteriaceae producers of class A and class C beta-lactamases. J. Environ. Sci. Health.

[119] Blaak H., Lynch G., Italiaander R., Hamidjaja R.A., Schets F.M., de Roda Husman A.M. (2015). Multidrug-resistant and extended spectrum Beta-lactamase-producing Escherichia coli in Dutch surface water and wastewater. PLoS ONE.

[120] Bouki C., Venieri D., Diamadopoulos E. (2013). Detection and fate of antibiotic resistant bacteria in wastewater treatment plants: A review. Ecotoxicol. Environ. Saf..

[121] Brechet C., Plantin J., Sauget M., Thouverez M., Talon D., Cholley P., Guyeux C., Hocquet D., Bertrand X. (2014). Wastewater treatment plants release large amounts of extended-spectrum beta-lactamase-producing Escherichia coli into the environment. Clin. Infect. Dis..

[122] Auerbach E.A., Seyfried E.E., McMahon K.D. (2007). Tetracycline resistance genes in activated sludge wastewater treatment plants. Water Res..

[123] Kim S., Park H., Chandran K. (2010). Propensity of activated sludge to amplify or attenuate tetracycline resistance genes and tetracycline resistant bacteria: A mathematical modeling approach. Chemosphere.

[124] McKinney C.W., Pruden A. (2012). Ultraviolet disinfection of antibiotic resistant bacteria and their antibiotic resistance genes in water and wastewater. Environ. Sci. Technol..

[125] Pruden A., Larsson D.G.J., Amézquita A., Collignon P., Brandt K.K., Graham D.W., Lazorchak J.M., Suzuki S., Silley P., Snape J.R. (2013). Management options for reducing the release of antibiotics and antibiotic resistance genes to the environment. Environ. Health Perspect..

[126] Hembach N., Schmid F., Alexander J., Hiller C., Rogall E.T., Schwartz T. (2017). Occurrence of the mcr-1 colistin resistance gene and other clinically relevant antibiotic resistance genes in microbial populations at different municipal wastewater treatment plants in Germany. Front. Microbiol..

[127] Gupta S.K., Shin H., Han D., Hur H.G., Unno T. (2018). Metagenomic analysis reveals the prevalence and persistence of antibiotic- and heavy metal-resistance genes in wastewater treatment plant. J. Microbiol..

[128] Marano R.B.M., Zolti A., Jurkevitch E., Cytryn E. (2019). Antibiotic resistance and class 1 integron gene dynamics along effluent, reclaimed wastewater irrigated soil, crop continua: Elucidating potential risks and ecological constraints. Water Res..

[129] Munir M., Wong K., Xagoraraki I. (2011). Release of antibiotic resistant bacteria and genes in the effluent and biosolids of five wastewater utilities in Michigan. Water Res..

[130] Rose J.B., Farrah S.R., Harwood V.J., Levine A.D., Lukaskik J., Menendez P., Scott T.M. (2004). Reduction of Pathogens, Indicator Bacteria, and Alternative Indicators by Wastewater Treatment and Reclamation Processes.

[131] Asano T., Burton F., Leverenz H., Tsuchihashi R., Tchobanoglous G. (2007). Water Reuse: Issues, Technologies, and Applications.

[132] Mitch A.A., Gasner K.C., Mitch W.A. (2010). Fecal coliform accumulation within a river subject to seasonally-disinfected wastewater discharges. Water Res..

[133] Rodriguez-Mozaz S., Vaz-Moreira I., Varela Della Giustina S., Llorca M., Barceló D., Schubert S., Berendonk T.U., Michael-Kordatou I., Fatta-Kassinos D., Martinez J.L. (2020). Antibiotic residues in final effluents of European wastewater treatment plants and their impact on the aquatic environment. Environ. Int..

[134] Gogoi A., Mazumder P., Tyagi V.K., Tushara Chaminda G.G., An A.K., Kumar M. (2018). Occurrence and fate of emerging contaminants in water environment: A review. Groundw. Sustain. Dev..

[135] Karthikeyan K.G., Meyer M.T. (2006). Occurrence of antibiotics in wastewater treatment facilities in Wisconsin, USA. Sci. Total Environ..

[136] Guo J., Li J., Chen H., Bond P.L., Yuan Z. (2017). Metagenomic analysis reveals wastewater treatment plants as hotspots of antibiotic resistance genes and mobile genetic elements. Water Res..

[137] Fatta-Kassinos D., Cytryn E., Donner E., Zhang T. (2020). Challenges related to antimicrobial resistance in the framework of urban wastewater reuse. Water Res..

[138] Li N., Sheng G.-P., Lu Y.-Z., Zeng R.J., Yu H.-Q. (2017). Removal of antibiotic resistance genes from wastewater treatment plant effluent by coagulation. Water Res..

[139] Guo J., Wang Y., Ahmed Y., Jin M., Li J., Manaia C.M., Donner E., Vaz-Moreira I., Hong P. (2020). Control strategies to combat dissemination of antibiotic resistance in urban water systems. Antibiotic Resistance in the Environment: A Worldwide Overview.

[140] Mantilla-Calderon D., Plewa M.J., Michoud G., Fodelianakis S., Daffonchio D., Hong P.Y. (2019). Water disinfection byproducts increase natural transformation rates of environmental DNA in Acinetobacter baylyi ADP1. Environ. Sci. Technol..

[141] Augsburger N., Mantilla-Calderon D., Daffonchio D., Hong P.-Y. (2019). Acquisition of extracellular DNA by Acinetobacter baylyi ADP1 in response to solar and UV-C254nm disinfection. Environ. Sci. Technol..

[142] Subirats J., Di Cesare A., Varela Della Giustina S., Fiorentino A., Eckert E.M., Rodriguez-Mozaz S., Borrego C.M., Corno G. (2019). High-quality treated wastewater causes remarkable changes in natural microbial communities and intI1 gene abundance. Water Res..

[143] U.S. Environmental Protection Agency (USEPA) (2005). Profile of the Healthcare Industry: EPA Office of Compliance Sector Notebook Project.

[144] U.S. Environmental Protection Agency (USEPA) (2010). NPDES Permit Writers’ Manual.

[145] U.S. Environmental Protection Agency (USEPA) (2000). Biosolids Technology Fact Sheet Land Application of Biosolids.

[146] Brooks J.P., Maxwell S.L., Rensing C., Gerba C.P., Pepper I.L. (2007). Occurrence of antibiotic-resistant bacteria and endotoxin associated with the land application of biosolids. Can. J. Microbiol..

[147] Munir M., Xagoraraki I. (2011). Levels of antibiotic resistance genes in manure, biosolids, and fertilized soil. J. Environ. Qual..

[148] Diehl D.L., LaPara T.M. (2010). Effect of temperature on the fate of genes encoding tetracycline resistance and the integrase of class 1 integrons within anaerobic and aerobic digesters treating municipal wastewater solids. Environ. Sci. Technol..

[149] Ma Y., Wilson C.A., Novak J.T., Riffat R., Aynur S., Murthy S., Pruden A. (2011). Effect of various sludge digestion conditions on sulfonamide, macrolide, and tetracycline resistance genes and class I integrons. Environ. Sci. Technol..

[150] Reinthaler F.F., Feierl G., Galler H., Haas D., Leitner E., Mascher F., Melkes A., Posch J., Winter I., Zarfel G. (2010). ESBL-producing E. coli in Austrian sewage sludge. Water Res..

[151] Huijbers P.M.C., Blaak H., de Jong M.C.M., Graat E.A.M., Vandenbroucke-Grauls C.M.J.E., de Roda Husman A.M. (2015). Role of the environment in the transmission of antimicrobial resistance to humans: A review. Environ. Sci. Technol..

[152] Neilson J.W., Josephson K.L., Pepper I.L., Arnold R.B., Di Giovanni G.D., Sinclair N.A. (1994). Frequency of horizontal gene transfer of a large catabolic plasmid (pJP4) in soil. Appl. Environ. Microbiol..

[153] U.S. Environmental Protection Agency (USEPA) (2013). Literature review of Contaminants in Livestock and Poultry Manure and Implications for Water Quality.

[154] Van Boeckel T.P., Pires J., Silvester R., Zhao C., Song J., Criscuolo N.G., Gilbert M., Bonhoeffer S., Laxminarayan R. (2019). Global trends in antimicrobial resistance in animals in low- and middle-income countries. Science.

[155] Animal Health Institute (AHI) (2000). Survey Indicates Most Antibiotics Used in Animals are Used for Treating and Preventing Disease.

[156] Done H.Y., Venkatesan A.K., Halden R.U. (2015). Does the recent growth of aquaculture create antibiotic resistance threats different from those associated with land animal production in agriculture?. AAPS J..

[157] Levy S.B. (1998). The challenge of antibiotic resistance. Sci. Am..

[158] Stockwell V.O., Duffy B. (2012). Use of antibiotics in plant agriculture. Rev. Sci. Technol..

[159] Florini K., Denison R., Stiffler T., Fitzgerald T., Goldburg R. (2005). Resistant Bugs and Antibiotic Drugs: State and County Estimates of Antibiotics in Agricultural Feed and Animal Waste.

[160] Hoelzer K., Wong N., Thomas J., Talkington K., Jungman E., Coukell A. (2017). Antimicrobial drug use in food-producing animals and associated human health risks: What, and how strong, is the evidence?. BMC Vet. Res..

[161] Peak N., Knapp C.W., Yang R.K., Hanfelt M.M., Smith M.S., Aga D.S., Graham D.W. (2007). Abundance of six tetracycline resistance genes in wastewater lagoons at cattle feedlots with different antibiotic use strategies. Environ. Microbiol..

[162] Sapkota A.R., Curriero F.C., Gibson K.E., Schwab K.J. (2007). Antibiotic-resistant enterococci and fecal indicators in surface water and groundwater impacted by a concentrated Swine feeding operation. Environ. Health Perspect..

[163] Bernot M.J., Smith L., Frey J. (2013). Human and veterinary pharmaceutical abundance and transport in a rural central Indiana stream influenced by confined animal feeding operations (CAFOs). Sci. Total Environ..

[164] Hubbard L.E., Givens C.E., Griffin D.W., Iwanowicz L.R., Meyer M.T., Kolpin D.W. (2020). Poultry litter as potential source of pathogens and other contaminants in groundwater and surface water proximal to large-scale confined poultry feeding operations. Sci. Total Environ..

[165] Graham J.P., Nachman K.E. (2010). Managing waste from confined animal feeding operations in the United States: The need for sanitary reform. J. Water Health.

[166] U.S. Environmental Protection Agency (USEPA) (2005). Detecting and Mitigating the Environmental Impact of Fecal Pathogens Originating from Confined Animal Feeding Operations: Review.

[167] Thanner S., Drissner D., Walsh F. (2016). Antimicrobial resistance in agriculture. MBio.

[168] Suttner B., Johnston E.R., Orellana L.H., Rodriguez R.L., Hatt J.K., Carychao D., Carter M.Q., Cooley M.B., Konstantinidis K.T. (2020). Metagenomics as a public health risk assessment tool in a study of natural creek sediments influenced by agricultural and livestock runoff: Potential and limitations. Appl. Environ. Microbiol..

[169] Abraham S., Sahibzada S., Hewson K., Laird T., Abraham R., Pavic A., Truswell A., Lee T., O’Dea M., Jordan D. (2020). Emergence of fluoroquinolone-resistant Campylobacter jejuni and Campylobacter coli among Australian chickens in the absence of fluoroquinolone use. Appl. Environ. Microbiol..

[170] Lerma L.L., Benomar N., Knapp C.W., Correa Galeote D., Galvez A., Abriouel H. (2014). Diversity, distribution and quantification of antibiotic resistance genes in goat and lamb slaughterhouse surfaces and meat products. PLoS ONE.

[171] Svanström P. (2014). Pathogens and Antibiotic Resistant Bacteria in Abattoir Waste and Animals—A Study Involving Abattoir Wastewater, Earthworms and Marabou Storks.

[172] Onuoha S. (2018). Distribution and antibiogram of bacterial species in effluents from abattoirs in Nigeria. J. Environ. Occup. Sci..

[173] Onuoha S., Okafor C., Aduo B., Nwaka F. (2016). Distribution of antibiotic resistant bacteria from abattoir wastes and its receiving waters at Nkwo-ezzamgbo, Ebonyi State, Nigeria. World J. Med. Sci..

[174] Afsharnia M., Naraghi B., Mardaneh J., Kianmehr M., Biglari H. (2018). The data of Escherichia coli strains genes in different types of wastewater. Data Brief.

[175] Burkhart K., Bernhardt C., Pelton T., Schaeffer E., Phillips A. (2018). Water Pollution from Slaughterhouses: Three Quarters of U.S. Meat Processing Plants that Discharge into Waterways Violated their Permits, 2016–2018.

[176] Chattopadhyay S. (2018). Exposure Assessment of Livestock Carcass Management Options during a Foreign Animal Disease Outbreak.

[177] U.S. Environmental Protection Agency (USEPA) (2017). Pesticide Registration: What are Antimicrobial Pesticides?.

[178] Kellogg R.L., Nehring R., Grube A., Goss D.W., Plotkin S. (2000). Environmental indicators of pesticide leaching and runoff from farm fields. Agric Prod.

[179] Burr T.J., Norelli J.L., Katz B., Wilcox W.F., Hoying S.A. (1988). Streptomycin resistance of Pseudomonas syringae pv. papulans in apple orchards and its association with a conjugative plasmid. Phytopathology.

[180] Rodríguez C., Lang L., Wang A., Altendorf K., García F., Lipski A. (2006). Lettuce for human consumption collected in Costa Rica contains complex communities of culturable oxytetracycline- and gentamicin-resistant bacteria. Appl. Environ. Microbiol..

[181] Rodríguez-Sánchez C., Altendorf K., Smalla K., Lipski A. (2008). Spraying of oxytetracycline and gentamicin onto field-grown coriander did not affect the abundance of resistant bacteria, resistance genes, and broad host range plasmids detected in tropical soil bacteria. Biol. Fertil. Soils.

[182] Yashiro E., McManus P.S. (2012). Effect of streptomycin treatment on bacterial community structure in the apple phyllosphere. PLoS ONE.

[183] Norelli J.L., Burr T.J., Lo Cicero A.M., Gilbert M.T., Katz B.H. (1991). Homologous streptomycin resistance gene present among diverse gram-negative bacteria in New York State apple orchards. Appl. Environ. Microbiol..

[184] U.S. Department of Agriculture (USDA) (2018). 2013 Census of Agriculture.

[185] Food and Agricultural Organization (FAO) (1989). Aquaculture Systems and Practices: A Selected Review.

[186] Watts J.E.M., Schreier H.J., Lanska L., Hale M.S. (2017). The rising tide of antimicrobial resistance in aquaculture: Sources, sinks and solutions. Mar. Drugs.

[187] Baquero F., Martínez J.-L., Cantón R. (2008). Antibiotics and antibiotic resistance in water environments. Curr. Opin. Biotechnol..

[188] Muziasari W.I., Parnanen K., Johnson T.A., Lyra C., Karkman A., Stedtfeld R.D., Tamminen M., Tiedje J.M., Virta M. (2016). Aquaculture changes the profile of antibiotic resistance and mobile genetic element associated genes in Baltic Sea sediments. FEMS Microbiol. Ecol..

[189] Reverter M., Sarter S., Caruso D., Avarre J.-C., Combe M., Pepey E., Pouyaud L., Vega-Heredía S., de Verdal H., Gozlan R.E. (2020). Aquaculture at the crossroads of global warming and antimicrobial resistance. Nat. Commun..

[190] Cole D., Drum D.J., Stalknecht D.E., White D.G., Lee M.D., Ayers S., Sobsey M., Maurer J.J. (2005). Free-living Canada geese and antimicrobial resistance. Emerg. Infect. Dis..

[191] Hower S., Phillips M.C., Brodsky M., Dameron A., Tamargo M.A., Salazar N.C., Jackson C.R., Barrett J.B., Davidson M., Davis J. (2013). Clonally related methicillin-resistant Staphylococcus aureus isolated from short-finned pilot whales (Globicephala macrorhynchus), human volunteers, and a bayfront cetacean rehabilitation facility. Microb. Ecol..

[192] Alm E.W., Daniels-Witt Q.R., Learman D.R., Ryu H., Jordan D.W., Gehring T.M., Santo Domingo J. (2018). Potential for gulls to transport bacteria from human waste sites to beaches. Sci. Total Environ..

[193] Dolejska M., Literak I. (2019). Wildlife is overlooked in the epidemiology of medically important antibiotic-resistant bacteria. Antimicrob. Agents Chemother..

[194] Dolejska M., Papagiannitsis C.C. (2018). Plasmid-mediated resistance is going wild. Plasmid.

[195] Ramey A.M., Ahlstrom C.A. (2020). Antibiotic Resistant Bacteria in Wildlife: Perspectives on Trends, Acquisition and Dissemination, Data Gaps, and Future Directions. J. Wildl. Dis..

[196] Vogt N.A., Stevens C.P.G., Pearl D.L., Taboada E.N., Jardine C.M. (2020). Generalizability and comparability of prevalence estimates in the wild bird literature: Methodological and epidemiological considerations. Anim. Health Res. Rev..

[197] Rogers S.W., Shaffer C.E., Langen T.A., Jahne M., Welsh R. (2018). Antibiotic-resistant genes and pathogens shed by wild deer correlate with land application of residuals. EcoHealth.

[198] Guenther S., Ewers C., Wieler L.H. (2011). Extended-spectrum beta-lactamases producing E. coli in wildlife, yet another form of environmental pollution?. Front. Microbiol..

[199] Sjolund M., Bonnedahl J., Hernandez J., Bengtsson S., Cederbrant G., Pinhassi J., Kahlmeter G., Olsen B. (2008). Dissemination of multidrug-resistant bacteria into the Arctic. Emerg. Infect. Dis..

[200] Fenlon D.R. (1981). Seagulls (Larus spp.) as vectors of salmonellae: An investigation into the range of serotypes and numbers of salmonellae in gull faeces. J. Hyg..

[201] Choi S., Chu W., Brown J., Becker S.J., Harwood V.J., Jiang S.C. (2003). Application of enterococci antibiotic resistance patterns for contamination source identification at Huntington Beach, California. Mar. Pollut. Bull..

[202] Fogarty L.R., Haack S.K., Wolcott M.J., Whitman R.L. (2003). Abundance and characteristics of the recreational water quality indicator bacteria Escherichia coli and enterococci in gull faeces. J. Appl. Microbiol..

[203] Simoes R.R., Poirel L., Da Costa P.M., Nordmann P. (2010). Seagulls and beaches as reservoirs for multidrug-resistant Escherichia coli. Emerg. Infect. Dis..

[204] Bonnedahl J., Drobni M., Gauthier-Clerc M., Hernandez J., Granholm S., Kayser Y., Melhus A., Kahlmeter G., Waldenstrom J., Johansson A. (2009). Dissemination of Escherichia coli with CTX-M type ESBL between humans and yellow-legged gulls in the south of France. PLoS ONE.

[205] Guenther S., Aschenbrenner K., Stamm I., Bethe A., Semmler T., Stubbe A., Stubbe M., Batsajkhan N., Glupczynski Y., Wieler L.H. (2012). Comparable high rates of extended-spectrum-beta-lactamase-producing Escherichia coli in birds of prey from Germany and Mongolia. PLoS ONE.

[206] Wade T.J., Calderon R.L., Brenner K.P., Sams E., Beach M., Haugland R., Wymer L., Dufour A.P. (2008). High sensitivity of children to swimming-associated gastrointestinal illness: Results using a rapid assay of recreational water quality. Epidemiology.

[207] Wade T.J., Sams E., Brenner K.P., Haugland R., Chern E., Beach M., Wymer L., Rankin C.C., Love D., Li Q. (2010). Rapidly measured indicators of recreational water quality and swimming-associated illness at marine beaches: A prospective cohort study. Environ. Health.

[208] O’Flaherty E., Solimini A., Pantanella F., Cummins E. (2019). The potential human exposure to antibiotic resistant-Escherichia coli through recreational water. Sci. Total Environ..

[209] Blackburn B.G., Craun G.F., Yoder J.S., Hill V., Calderon R.L., Chen N., Lee S.H., Levy D.A., Beach M.J. (2004). Surveillance for waterborne-disease outbreaks associated with drinking water—United States, 2001–2002. MMWR Surveill Summ..

[210] Grisey E., Belle E., Dat J., Mudry J., Aleya L. (2010). Survival of pathogenic and indicator organisms in groundwater and landfill leachate through coupling bacterial enumeration with tracer tests. Desalination.

[211] Gerba C.P. (2000). Assessment of enteric pathogen shedding by bathers during recreational activity and its impact on water quality. Quant. Microbiol..

[212] Huijbers P.M.C., Flach C.-F., Larsson D.G.J. (2019). A conceptual framework for the environmental surveillance of antibiotics and antibiotic resistance. Environ. Int..

[213] Chiao T.H., Clancy T.M., Pinto A., Xi C., Raskin L. (2014). Differential resistance of drinking water bacterial populations to monochloramine disinfection. Environ. Sci. Technol..

[214] Knight G.M., Davies N.G., Colijn C., Coll F., Donker T., Gifford D.R., Glover R.E., Jit M., Klemm E., Lehtinen S. (2019). Mathematical modelling for antibiotic resistance control policy: Do we know enough?. BMC Infect. Dis..

[215] Ashbolt N.J., Amezquita A., Backhaus T., Borriello P., Brandt K.K., Collignon P., Coors A., Finley R., Gaze W.H., Heberer T. (2013). Human Health Risk Assessment (HHRA) for environmental development and transfer of antibiotic resistance. Environ. Health Perspect..

[216] Amos G.C.A., Gozzard E., Carter C.E., Mead A., Bowes M.J., Hawkey P.M., Zhang L., Singer A.C., Gaze W.H., Wellington E.M.H. (2015). Validated predictive modelling of the environmental resistome. ISME J..

[217] Gillings M.R., Gaze W.H., Pruden A., Smalla K., Tiedje J.M., Zhu Y.G. (2015). Using the class 1 integron-integrase gene as a proxy for anthropogenic pollution. ISME J..

[218] Ovejero C.M., Delgado-Blas J.F., Calero-Caceres W., Muniesa M., Gonzalez-Zorn B. (2017). Spread of mcr-1-carrying Enterobacteriaceae in sewage water from Spain. J. Antimicrob. Chemother..

[219] Wang R., van Dorp L., Shaw L.P., Bradley P., Wang Q., Wang X., Jin L., Zhang Q., Liu Y., Rieux A. (2018). The global distribution and spread of the mobilized colistin resistance gene mcr-1. Nat. Commun..

[220] Pruden A., Arabi M., Storteboom H.N. (2012). Correlation between upstream human activities and riverine antibiotic resistance genes. Environ. Sci. Technol..

[221] He L.Y., Liu Y.S., Su H.C., Zhao J.L., Liu S.S., Chen J., Liu W.R., Ying G.G. (2014). Dissemination of antibiotic resistance genes in representative broiler feedlots environments: Identification of indicator ARGs and correlations with environmental variables. Environ. Sci. Technol..

[222] Vital P.G., Zara E.S., Paraoan C.E.M., Dimasupil M., Angela Z., Abello J.J.M., Santos I.T.G., Rivera W.L. (2018). Antibiotic resistance and extended-spectrum Beta-Lactamase production of Escherichia coli isolated from irrigation waters in selected urban farms in metro Manila, Philippines. Water.

[223] Hill R., Jahne M., Keely S., Brinkman N., Haugland R., Leibowitz S., Wheaton E., Garland J., Martin R. (2019). Modeling and Predicting the Occurrences of Antibiotic Resistance Genes in US Rivers and Streams.

[224] Stachler E., Crank K., Bibby K. (2019). Co-occurrence of crAssphage with antibiotic resistance genes in an impacted urban watershed. Environ. Sci. Technol. Lett..

[225] Li J., Cheng W., Xu L., Strong P.J., Chen H. (2015). Antibiotic-resistant genes and antibiotic-resistant bacteria in the effluent of urban residential areas, hospitals, and a municipal wastewater treatment plant system. Environ. Sci. Pollut. Res. Int..

[226] Pholwat S., Liu J., Taniuchi M., Chinli R., Pongpan T., Thaipisutikul I., Ratanakorn P., Platts-Mills J.A., Fleece M., Stroup S. (2019). Genotypic antimicrobial resistance assays for use on E. coli isolates and stool specimens. PLoS ONE.

[227] McDermott P.F., Tyson G.H., Kabera C., Chen Y., Li C., Folster J.P., Ayers S.L., Lam C., Tate H.P., Zhao S. (2016). Whole-genome sequencing for detecting antimicrobial resistance in nontyphoidal Salmonella. Antimicrob. Agents Chemother..

[228] Centers for Disease Control and Prevention (CDC) (2020). National Antimicrobial Resistance Monitoring System (NARMS) Now: Human Data.

[229] Matheu J., Aidara-Kane A., Andremont A. (2017). The ESBL Tricycle AMR Surveillance Project: A Simple, One Health Approach to Global Surveillance.

[230] World Health Organization (WHO) (2018). Global Antimicrobial Resistance Surveillance System (GLASS) Report: Early Implementation 2017–2018.

[231] World Health Organization (WHO) (2019). Monitoring and Evaluation of the Global Action Plan on Antimicrobial Resistance: Framework and Recommended Indicators.

